# Unpacking Performance Factors of Innovation Systems and Studying Germany’s Attempt to Foster the Role of the Patient Through a Market Access Pathway for Digital Health Applications (DiGAs): Exploratory Mixed Methods Study

**DOI:** 10.2196/66356

**Published:** 2025-01-06

**Authors:** Sara Gehder, Moritz Goeldner

**Affiliations:** 1 Working Group for Data-Driven Innovation Hamburg University of Technology Hamburg Germany

**Keywords:** regulatory market access pathways, digital health application, DiGA, patient-relevant structural and procedural improvement, pSVV pathway analysis, qualitative and systemic analysis, policy and stakeholder insights, innovation system analysis

## Abstract

**Background:**

Health care innovation faces significant challenges, including system inertia and diverse stakeholders, making regulated market access pathways essential for facilitating the adoption of new technologies. The German Digital Healthcare Act, introduced in 2019, offers a model by enabling digital health applications (DiGAs) to be reimbursed by statutory health insurance, improving market access and patient empowerment. However, the factors influencing the success of these pathways in driving innovation remain unclear.

**Objective:**

This study aims to identify the key performance factors of the innovation system shaped by the *patient-relevant structural and procedural improvement* (pSVV) pathway within the DiGA model. By examining how this pathway supports the entry of innovative digital health technologies, we seek to uncover the systemic dynamics that influence its effectiveness in fostering patient-centered digital health solutions.

**Methods:**

This study, conducted from May 2023 to November 2024, used a mixed methods approach. A descriptive analysis assessed how DiGA manufacturers use positive health care effects, giving a market overview of the pSVV technology. A qualitative analysis using grounded theory and Gioia methodology provided insights into stakeholder perspectives, focusing on manufacturers and regulatory bodies. A functional-structural analysis examined how components of the innovation system, such as actors, institutions, interactions, and infrastructure, interact and impact the effectiveness of the pathway.

**Results:**

The descriptive analysis showed that only 11 (20%) of the 56 DiGAs available in Germany used the pSVV pathway, with only 1 (2%) provisionally listed DiGA using pSVV as a primary end point; 6 of 9 (67%) pSVV key areas were used. The qualitative analysis revealed that manufacturers prioritize demonstrating medical benefits over pSVV due to evidence requirements and uncertainties around pSVV acceptance. Operational barriers hindered the adoption of pSVV, despite a positive reception among stakeholders. The systemic analysis identified key issues, including a lack of entrepreneurial focus on pSVV, limited regulatory experience, inadequate measurement methods, and entrenched practices prioritizing medical benefits, that hinder market formation and legitimacy.

**Conclusions:**

This study identifies key factors for effectively implementing innovation systems through regulated market access pathways, including content and format security, clearer framework specification, active innovation process management, and market formation stimulation. Addressing these factors can reduce uncertainties and promote wider adoption of digital health technologies. The findings highlight the need for future research on patient empowerment and the development of methodologies beyond traditional therapeutic outcomes.

## Introduction

### Background

Innovation in health care is well known for being challenging. The complexity is driven by factors such as the involvement of diverse stakeholders in highly regulated markets [1,2], the need to address “wicked problems,” complex issues without straightforward solutions [3], and substantial “system inertia,” meaning resistance to change, which is especially pronounced in health care systems [4]. Policy approaches have been shown to act as inertial forces that can significantly shape the development of innovative technologies in health care [5], particularly digital health technologies, where patient access is greatly limited without broad and scalable market access pathways [6]. Mobile health apps are becoming increasingly available to consumers worldwide [7-9]; however, significant barriers, including regulatory complexity, insufficient evidence of clinical benefit, and data privacy concerns, still prevent their full integration into health care systems, leaving them underused in clinical practice [10-14].

To address these challenges, health policy approaches are being adopted globally to standardize regulatory processes and speed up market access, particularly in the rapidly evolving field of digital health [15-18]. Notable examples of health policy approaches include regulated market access pathways, such as *the fast-track process for digital health applications* pathway in Germany [19] and the *Prise en Charge Anticipée Numérique* (PECAN) pathway in France [20], which aim to facilitate the entry of digital health applications (DiGAs; German: Digitale Gesundheitsanwendung) into their respective markets. These new approaches are gaining significant international attention as pioneering models in the field [6,21].

To illustrate, the German Digital Healthcare Act (DVG), introduced in 2019, established the DiGA pathway to foster the integration of so-called DiGAs into the health care system. This pathway allows DiGAs to be reimbursed by statutory health insurance when prescribed by health care professionals or provided by health insurers. With approximately 90% of the German population covered by statutory health insurance [22], these “apps on prescription” are widely accessible at no cost to patients. Eligible DiGAs are included in the national DiGA Directory [23], which is managed by the Federal Institute for Drugs and Medical Devices (BfArM; German: Bundesinstitut für Arzneimittel und Medizinprodukte) to ensure transparency and oversee the approval process [24,25]. To qualify, a DiGA must be Conformité Européenne (CE)–marked (classes I and IIa or IIb), be based primarily on digital technology intended for direct patient use, and support the detection, monitoring, treatment, or alleviation of diseases, injuries, or disabilities [26]. Following a recent regulatory update in April 2024, digital medical devices classified as class IIb have been added to be eligible for recognition as a DiGA. However, this inclusion comes with specific stipulations; these devices are required to demonstrate medical benefit only, and they are eligible solely for permanent listing, excluding the option for preliminary listing. In addition, a DiGA must meet stringent requirements for safety, quality, functionality, privacy, and data security as defined in the respective DiGA ordinance (DiGAV; German: Digitiale Gesundheitsanwendungen-Verordnung) [24]. To enhance transparency around the assessment criteria and specific requirements established by BfArM, a DiGA guide [27] for manufacturers, service providers, and users was published. This guide provides a concise overview of the applicable ordinances and regulations, detailing how BfArM interprets the normative requirements set forth in the DVG and DiGAV.

Despite some emerging research examining the market dynamics following the implementation of regulated market access pathways, such as the number of products entering the market, time to market, pricing, and study designs used to demonstrate efficacy [28,29], as well as factors contributing to the success of specific DiGA products [30], broader factors influencing successful technology integration remain underexplored. This study addresses these gaps to better inform future policy and innovation strategies. To identify potential success factors, it is crucial to consider not only the regulated market access pathway itself but also the surrounding elements that influence its effectiveness. The broader network of actors, institutions, and resources involved in the creation, diffusion, and use of new technologies is defined as the technology innovation system [31]. A health policy approach, such as the creation of a regulated market access pathway that “brings about processes of change and stimulates technological innovation,” can be characterized as a systemic instrument [32]. Systemic instruments are mechanisms designed to shape the conditions under which technological innovation systems operate. Understanding the dynamics that a systemic instrument brings to a technological innovation system requires the consideration of various functional and structural components, all of which must be in place to effectively implement the targeted technological innovation [33]. These components, as outlined by Wieczorek and Hekkert [34], include the structural components, such as actors, institutions, interactions, and infrastructure, as well as the functional components, such as entrepreneurial activities (F1), knowledge development (F2), knowledge diffusion (F3), guidance of the search (F4), market formation (F5), mobilization of resources (F6), and creation of legitimacy (F7) [35].

This exploratory study aimed to identify factors contributing to the success of an innovation system shaped by a regulated market access pathway in the digital health sector. Through an exemplary case study, we sought insights to support ongoing improvements in digital health policy, focusing on optimizing market access pathways for digital health technologies. The central research question is as follows: What are the performance factors of an innovation system created by a regulated market access pathway that supports the market entry of innovative digital health technologies?

To address this question, we selected a specific case from the 2019 German policy innovation DVG, particularly the “patient-relevant structural and procedural improvements” (pSVV; German: Patientenrelevante Struktur- und Verfahrensverbesserung) pathway. The pSVV pathway is a targeted segment within the DiGA pathway, specifically modifying the element of product value assessment, 1 of the 4 “full-stack” elements (regulatory authorization, product value assessment, pricing and reimbursement, and patient access) that define a comprehensive market access pathway [6], promoting innovations aimed to specifically elevate DiGAs that enhance patient–health care provider interactions and support patient health behaviors.

Unlike traditional pharmaceuticals, which require evidence of “therapeutic efficacy” through clinical studies for coverage eligibility [36], DiGAs are allowed to demonstrate their effectiveness through “positive health care effects” defined as either “medical benefit” or pSVV. While the medical benefit criterion focuses on outcomes, such as improved health status, reduced disease duration, prolonged survival, or improved quality of life, the pSVV criterion introduces 9 additional areas to demonstrate the value that is equally eligible as defined in the DiGA guide. These areas include improving care coordination, adherence to guidelines and standards, enhancing patient adherence, facilitating access to care, increasing patient safety, and promoting health literacy and autonomy. This expansion of the traditional product value assessment beyond the traditional therapeutic benefits is further complemented in the DiGA guide by explicitly broadening the acceptable methods of evidence beyond the traditional randomized controlled trials. We selected this case due to the maturity of the evolving DiGA ecosystem [28] in Germany, which offers greater transparency and availability of data on use and uptake compared to other countries. Within this context, the pSVV pathway was chosen as a focal point because it represents a well-defined component within the DiGA pathway, involving a manageable number of stakeholders while offering sufficient accumulated experience to provide valuable insights into the factors influencing digital health innovation.

In addition, the introduction of the pSVV pathway is groundbreaking as it extends beyond traditional treatment parameters, embracing a holistic approach to health care that prioritizes patient empowerment and streamlined care processes [19,37,38]. This aligns with the global trend toward patient empowerment, emphasizing the importance of patient agency and involvement in health care processes [16,39,40].

Our research used a mixed methods approach to provide a comprehensive exploration of the factors at play. We began with a descriptive analysis of the innovation success of the targeted pSVV technology, which helps contextualize the case and assess the effectiveness of the policy intervention [41]. Following this, a qualitative analysis of the perception and use of the market access pathway was conducted to develop theoretical insights into the factors that influence the adoption of specific innovation pathways [42,43]. In addition, a functional-structural analysis of the technological innovation system was performed to uncover broader factors beyond the pathway itself that are essential for successful technological innovation [34].

### Contribution

This study contributes to the field by providing a comprehensive analysis of factors that support the successful integration of digital health innovations. Our findings will provide policy makers and stakeholders worldwide with practical insights to improve regulated market access pathways and drive innovation strategies that better meet the needs of health care systems and patient empowerment.

## Methods

### Setting and Study Design

This exploratory study used a mixed methods approach to investigate factors influencing the success of a regulated market access pathway for digital health technologies. We conducted a descriptive analysis to assess the adoption of pSVV technology, followed by a qualitative analysis to explore stakeholder perspectives on the pSVV pathway, using grounded theory and Gioia methodology to ensure neutrality and objectivity. Finally, we performed a functional-structural analysis to identify additional success factors within the broader technology innovation system. Each method is described in detail subsequently.

This study was carried out between May 2023 and November 2024 with interviews being conducted between May 2023 and November 2023.

First, a descriptive analysis of the use of positive health care effects by DiGA manufacturers was conducted to establish an understanding of the current developmental status of the pSVV technology in the market.

Second, a qualitative analysis was performed to explore the success factors of a regulated market access pathway by subsequently analyzing the perception and use of the pSVV pathway of key stakeholders. Since the pSVV pathway was only introduced in 2019 and has not been studied yet, a qualitative research approach was adopted. This approach allows for deep exploration of complex and less understood topics [44]. Unlike quantitative methods, which rely on larger sample sizes and predefined variables, qualitative methods provide flexibility for exploring nuanced, context-specific insights, particularly valuable given the relatively small number of relevant stakeholders (35 DiGA manufacturers as of early 2023). By using the inductive grounded theory method [45] alongside the Gioia methodology [42,43], we objectively analyzed the views of market participants on the pSVV pathway, avoiding any imposition of preconceived theories and ensuring neutrality in the analysis. For the qualitative analysis, we followed the Consolidated Criteria for Reporting Qualitative Research-32 (COREQ-32) checklist [46].

Building on the dataset from the qualitative analysis, we conducted a functional-structural analysis of the technology innovation system established by the pSVV pathway to identify additional success factors related to regulated market access pathways. This analysis followed the systemic policy framework established by Wieczorek and Hekkert [34], enabling a comprehensive examination of the innovation system’s functional dynamics and structural components within the current policy context.

While this study provided valuable insights into potential strategies to improve the pSVV pathway in particular, the international focus of this study precluded detailed insights into Germany-specific factors. To address this, we conducted a subsequent analysis focusing specifically on the question, “What factors facilitate or hinder the implementation of pSVV within the approval process for DiGA?” and “What specific requirements and expectations of relevant stakeholders must be addressed to establish the concept of pSVV in the market?” The results of this specific analysis were published in a German-language journal to inform stakeholders in this context [47].

### Sampling and Data Collection

For the execution of our descriptive analysis regarding the state of adoption of pSVV, we evaluated publicly available data on the positive health care effects of DiGA from the DiGA directory [23]. The data were retrieved via an application programming interface provided by BfArM for scientific use.

In our qualitative study, we used a hybrid approach of criterion sampling and snowball sampling for participant recruitment. We aimed to engage with stakeholders who have a direct influence on which digital health products enter the market and, if entry occurs through the DiGA pathway, determine the associated positive health care effects. The inclusion criteria are defined subsequently.

The first inclusion criterion was organizations that are directly involved with the DiGA ordinances and regulations, whether developing it, ensuring or supporting its implementation, or actively using it.

The second criterion was individuals, within these organizations, whose professional roles directly involve the development, implementation, or use of the DiGAVs and regulations.

The third inclusion criterion was additionally individuals who have actively decided against using the DiGA pathway, provided they have extensively engaged with the ordinances and regulations and are potentially qualified to use it (eg, manufacturers of DiGAs not listed as DiGA).

Individuals without direct and active influence over the selection of products entering the DiGA market or the choice of the positive health care effect were excluded.

Consequently, the exclusion criteria led to the exclusion of stakeholder groups in the DiGA market, such as DiGA users, prescribers, and researchers in the field, while the inclusion criteria led to the identification of the stakeholder groups presented in Textbox 1.

**Stakeholder groups included in qualitative analysis.**

- Digital health application (DiGA) manufacturers with patient-relevant structural and procedural improvement (pSVV; DiGA with pSVV): This category includes decision makers from manufacturers of DiGAs that are listed in the DiGA directory and have implemented pSVV as a positive health care effect.
- DiGA manufacturers without pSVV (DiGA without pSVV): This category comprises manufacturers who are listed in the DiGA directory but have not used pSVV as a positive health care effect.
- Manufacturers of mobile health applications not listed in the DiGA directory (non-DiGA): This group consists of manufacturers whose CE–certified mobile health apps are not, or are not yet, listed in the DiGA directory.
- Consultants: This group refers to professionals from advisory firms who specialize in assisting manufacturers in the process of getting listed in the DiGA directory.
- Regulatory bodies: This group encompasses public institutions that are deeply involved in the development and management of the DiGA pathway and the evolution of the guiding ordinances and regulations (Digital Healthcare Act [DVG], DiGA ordinance [DiGAV], and DiGA guide).

Due to the very small size of stakeholder groups 1 and 5, yet their significant relevance to our research questions, we contacted all organizations in these groups in accordance with our inclusion criteria. For groups 2 to 4, given the novelty and niche nature of the topic, we opted for snowball sampling [48] to achieve the highest possible participation rate. Individuals were excluded if someone from the same organization had already participated in the study, to avoid multiple responses from the same context. This combined approach resulted in a 66% positive response rate to our participation invitations. Participant recruitment began with initial email contacts, followed by referrals from existing participants in line with snowball sampling. Efforts were focused on ensuring a diverse representation of the identified stakeholder groups.

Our outreach targeted 29 individuals, with 19 (66%) agreeing to participate. Reasons for nonparticipation included lack of response (n=9, 31%) and time constraints (n=1, 4%). The distribution of speakers across groups and their organizational roles are detailed in Table S1 in Multimedia Appendix 1.

For the interviews, a semistructured approach was used with decision makers who had consented to participate and endorsed a data protection declaration. Efforts were made to ensure neutrality, with no prior relationships between interviewers and participants. An interview guide with 7 open-ended questions was prepared to facilitate conversation while ensuring neutrality and objectivity in the analysis. The themes of the questions were developed after preliminary data gathering, which included limited literature research and initial conversations with 2 experts in the field (a managing director from a manufacturer’s association and a member of an organization that has 1 DiGA listed in the DiGA directory), adhering to the standards of the Gioia methodology [42]. These experts were not part of the subsequent interviews, ensuring that the development of the interview guide was informed yet independent of the interview process itself (Multimedia Appendix 1). In total, 2 central themes emerged from this process: the general approval process for DiGA and the specifics of the pSVV pathway. For each of these themes, we formulated specific questions, which were refined during the initial interviews to ensure clarity and neutrality. Interviews were scheduled at the convenience of the participants and conducted via Zoom (Zoom Video Communications, Inc), with only the participant and the primary interviewer (SG) present, though 3 selected interviews had a second researcher (MG) present for quality assurance. No interviews were repeated.

Conducted in German, interviews lasted 25 to 35 minutes, audio tracks were recorded, transcribed using the software Whisper Transcription (Good Snooze), and anonymized. Data saturation was reached when no new first-order concepts or second-order themes emerged during analysis, as reflected in field notes and initial coding. This indicated that the data structure, developed through the Gioia methodology, was comprehensive, and additional data collection would not yield further insights. Following the analysis, data triangulation was enhanced through additional discussions with 4 experts from regulatory and digital health sectors to refine the results. This included a conversation with leading representatives from BfArM, which, due to confidentiality agreements, cannot be directly cited in this study. Insights from these discussions were used to cross-verify emerging themes and validate our interpretations, ensuring robustness in our conclusions.

### Ethical Considerations

Informed consent was obtained from all participants who were interviewed strictly as representatives of their respective employers. Participants were selected based solely on their relevance to the research question and their experience in the field, with no consideration of personal or socially sensitive attributes. No personal data or socially relevant information were collected, and all data were fully anonymized to ensure privacy and confidentiality. No compensation was provided for participation in the study.

Furthermore, the study did not involve vulnerable populations, health care professionals, medical procedures, health-related data, or any form of deception, manipulation, or intervention that could impact participants’ well-being. In addition, there was no risk of physical, psychological, social, or economic harm to participants. Following consultation with the head of the Institute for Ethics in Technology at Hamburg University of Technology and a review by the German Association for Experimental Economic Research eV (certificate number kNjYR7Ag), it was confirmed that specific ethics approval was not required. This approach fully aligns with best practices for ensuring ethical compliance in socioeconomic research.

### Descriptive Analysis

For this analysis, we included all DiGA listed in the DiGA directory at BfArM as of November 8, 2024. This study specifically aimed to compare how often medical benefits are used versus pSVV, as published in the DiGA directory [23]. To further refine our understanding of the adoption levels of pSVV, we also examined the use rates across the 9 key areas of pSVV detailed in the DiGA guide [27].

### Qualitative Analysis

Transcripts were methodically analyzed in a 3-step procedure using the grounded theory and the well-established Gioia methodology [42,49] to ensure qualitative rigor in inductive research. After familiarization with the primary data, a codebook was developed by generating codes in an iterative process using the software MaxQDA (VERBI Software). SG coded all (19/19, 100%) interviews, and 2 (11%) randomly selected interviews were cocoded by MG. In addition, SG and MG regularly discussed and reflected on the data collection process and preliminary findings throughout all phases of the research. A consensus on all codes was eventually reached by the research team.

After agreeing on a coding scheme that best captured the diversity of the material, the transcripts and codes were analyzed again to identify second-order categories. First-order concepts included concepts, such as “lack of methods is the biggest hurdle for proof of pSVV” or “pSVV good ideas but difficult to operationalize.” First-order concepts were combined into second-order themes, such as “study design for pSVV difficult due to lack of measurement instruments.” Ultimately, second-order themes were merged hierarchically into five aggregate dimensions that best summarize the theoretical contribution in the data: (1) “key aspects for manufacturers within the regulated market access pathway,” (2) “key aspects for study design,” (3) “reasons for considering possibilities within new pathway,” (4) “hurdles for considering possibilities within new pathway,” and (5) “targeted innovative technology important for the health care system” (refer to Figure S1 and Table S3 in Multimedia Appendix 1).

Following the analysis, a triangulation of the results was conducted with 4 individuals from institutions involved in the process: 1 interview participant and 3 additional experts with extensive subject matter expertise, who had not participated in the initial interview study. All 4 experts were selected from the regulatory field, as many of the derived implications pertained to regulatory issues.

### Functional-Structural Analysis

The first-order concepts, originally derived from the previous qualitative analysis as elucidated earlier, were subject to an additional analysis using the functional-structural systemic problem analyses framework [34], a methodology specifically tailored for the exploration of technology innovation systems [50,51]. Our analytical focus centered on the innovative pSVV technology, concentrating on the first-order concepts that laid the foundation for dimensions 2 to 5. Notably, the first dimension, “Key aspects for manufacturers within the regulated market access pathway,” was excluded from this analysis, as it did not provide insights specific to the pSVV aspects of the DiGA pathway, but rather addressed the pathway in general.

In step 1, to facilitate a comprehensive analysis, all first-order concepts corresponding to dimensions 2 to 5 were systematically organized into a 4×7 matrix. This matrix integrates the 4 structural elements and 7 functional dimensions of an innovation system [34,50,52]. Following the analysis framework, the objective was to elucidate overarching “systemic problems” hindering the intended innovation within the innovation system for pSVV. Refer to Table S4 in Multimedia Appendix 1 for the comprehensive assignment of concepts to the functional and structural dimensions of the innovation system and the derivation of “systemic problems.” The sorting of concepts, derivation of instruments, and clustering into strategic factors were conducted by SG and subsequently agreed upon by the research team.

In step 2, to address the identified “systemic problems,” we first identified “systemic instruments” within the functional-structural framework of Wieczorek and Hekkert [34], tailored to specific systemic issues.

In step 3, we organized the “systemic instruments” identified in step 2 into 3 strategic factors to form actionable clusters. A detailed overview of the derivation process is available in Table S5 in Multimedia Appendix 1.

## Results

### Current State of pSVV Adoption in the DiGA Market: Descriptive Analysis

As of November 8, 2024, out of the 56 DiGAs available in Germany, only 1 (2%) provisionally listed DiGA uses pSVV as its primary end point, with none in the permanently listed category. While 10 (19%) DiGAs incorporate pSVV as a secondary end point, the predominant focus remains on traditional medical benefits. The adoption of pSVV in comparison to medical benefits stands at 11 versus 45, meaning only 20% (11/56) of all currently listed DiGAs use pSVV. Moreover, only 1 (2%) of 56 DiGA exclusively focuses on pSVV as a primary end point [23].

**Figure 1:**
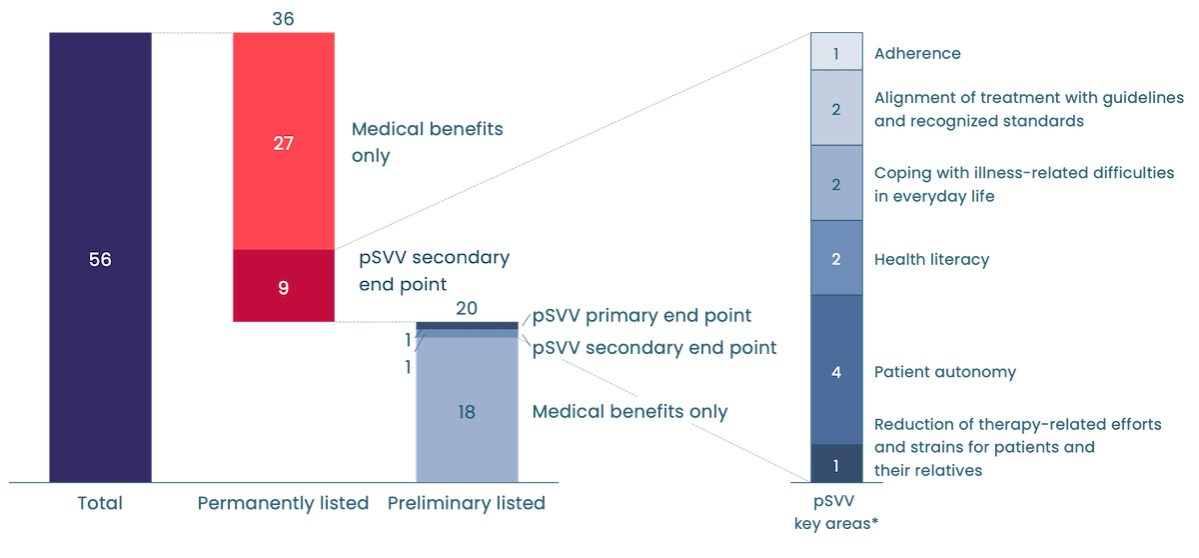
The number of digital health applications (DiGAs) listed in the DiGA directory in Germany based on positive health care effects and status of listing (as of November 8, 2024), including the breakdown of specific key areas used within patient-relevant structural and procedural improvements (pSVV). *Eleven DiGAs using pSVV use 12 pSVV end points with 1 DiGA using 2 pSVV key areas.

The 11 (20%) of the 56 DiGA currently listed that use pSVV as a primary or secondary end point have collectively used 12 pSVV end points (Figure 1). Among these, 6 (67%) of the 9 possible key areas outlined in the DiGA guide have been used. In total, 4 (44%) of these areas have been used more than once, namely “patient autonomy,” “health literacy,” “coping with disease-related difficulties in everyday life,” and “alignment of treatment with guidelines and recognized standards.”

The 11 (20%) of the 56 DiGA currently listed that use pSVV are owned by 10 organizations, with 1 organization using pSVV end points in clinical trials to prove the positive care effect for 2 of their DiGA products. We estimated the timing of the adoption of the pSVV pathway by examining the time frame of clinical studies and found that 2 DiGA demonstrating a pSVV had already been conducting their clinical studies before the pathway was introduced in 2019 (velibra and vorvida, both developed by GAIA AG).

### Analyzing Perception and Use of the pSVV Pathway by Key Stakeholders: Qualitative Analysis

#### Overview

**Figure 2:**
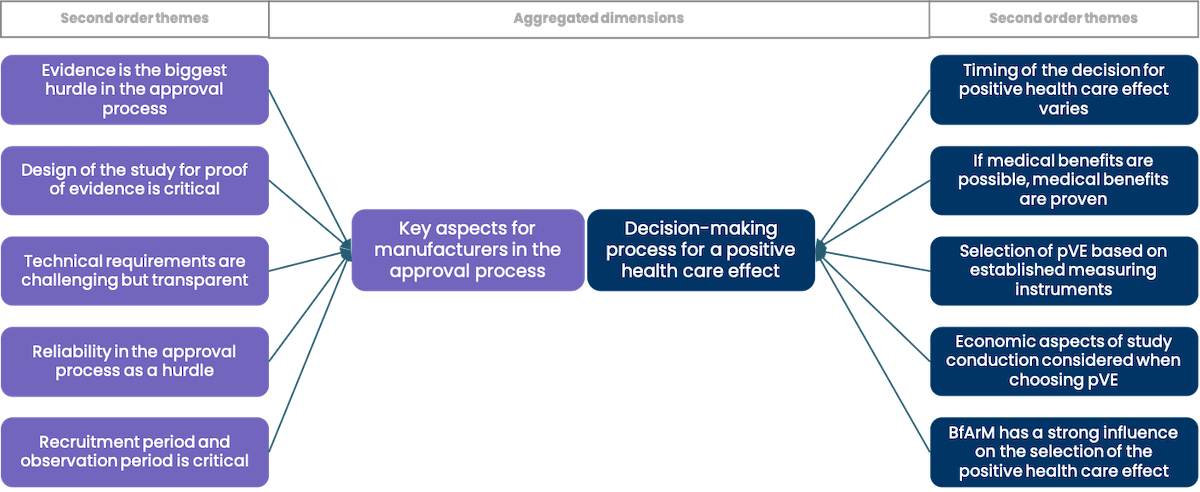
Graphical illustration of selected parts of the data structure derived from Gioia methodology illustrating key aspects for manufacturers within the regulated market access pathway as well as the key aspects of choosing the health care effect. BfArM: Federal Institute for Drugs and Medical Devices; pVE: positive health care effects.

**Figure 3:**
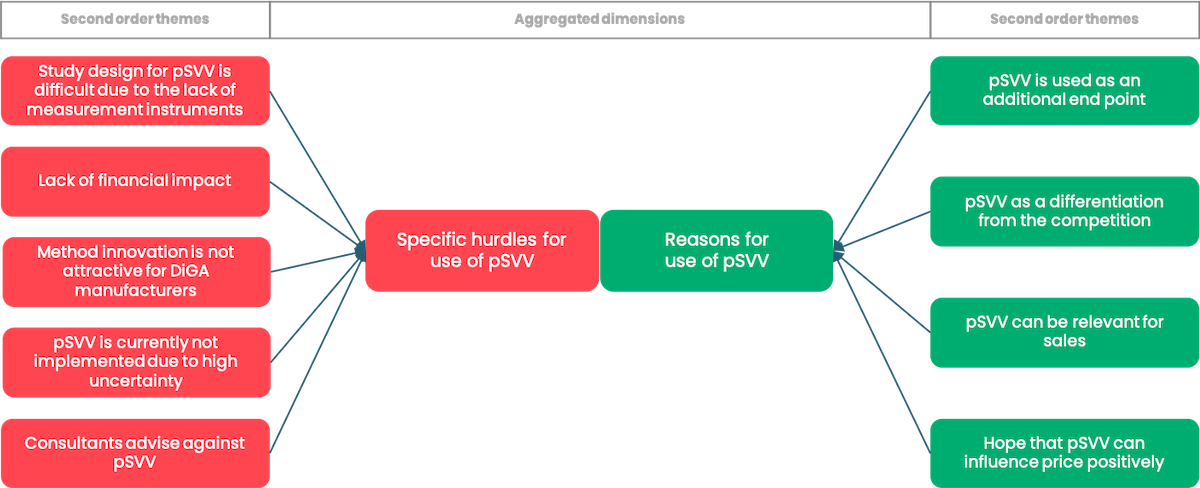
Graphical illustration of selected parts of the data structure derived from Gioia methodology illustrating hurdles of DiGA manufacturers for considering possibilities within the new pathway as well as reasons for doing so. DiGA: digital health application; pSVV: patient-relevant structural and procedural improvement.

**Figure 4:**
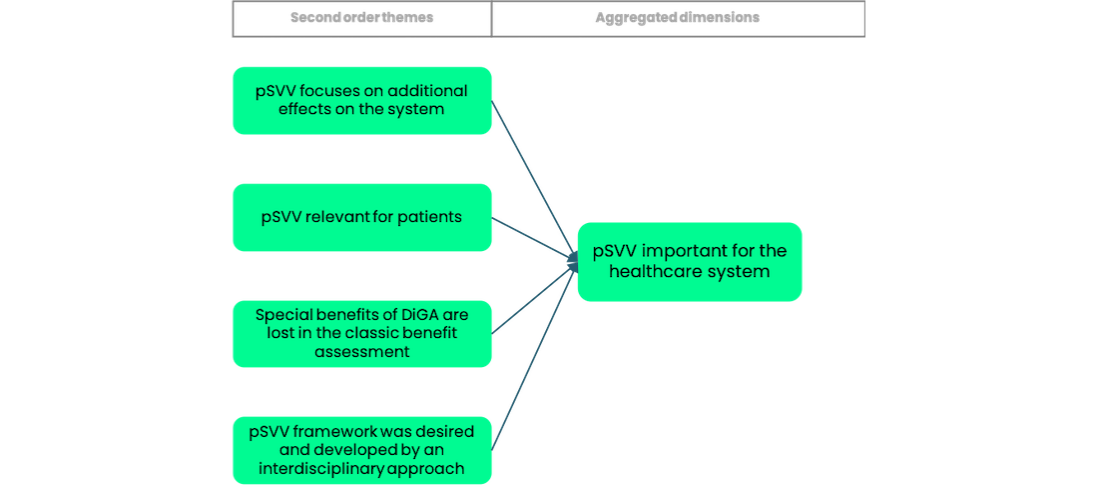
Graphical illustration of a selected part of the data structure derived from Gioia methodology illustrating the perception of the importance of patient-relevant structural and procedural improvements (pSVVs) in the health care system. DiGA: digital health application.

The results are presented based on the data structure developed through the Gioia methodology (Figure S1 in Multimedia Appendix 1), which organizes findings into first-order concepts, second-order themes, and overarching dimensions. In the subsequent sections, findings are systematically presented separating the data structure into 3 segments of aggregated dimensions and further detailed by their corresponding second-order themes. To illustrate our approach, Figures 2-4 provide a visual representation of the respective parts of the data structure. In addition, Table S3 in Multimedia Appendix 1 includes selected codes that were instrumental in forming the data structure.

#### Key Considerations in the Approval Process for DiGAs: Evidence as the Key Aspect

Being asked about the key considerations a DiGA manufacturer needs to face during the approval process, most (14/16, 88%) of the surveyed manufacturers and consultants concurred on a central theme—evidence takes the forefront, serving as the primary concern for manufacturers as “...(evidence) is the crucial point, which often determines the inclusion or non-inclusion (in the DiGA Directory)” (Consultant_5). In conjunction with this, the DiGA study design for evidence generation is deemed critical. The execution of the study is recognized as one of the primary cost drivers for manufacturers and the approving authority (BfArM) has a strong influence on the final design, as described by a participant, “And one can clearly see that an evidence level is crystallizing, which, within the framework that is possible, is already on the strictest side” (DiGA without pSVV_4).

While technical requirements, such as data privacy, interoperability, and accessibility were also mentioned, discussions in these domains with the BfArM were described as negligible due to the “clear and transparent” (Consultant_2) nature of the requirements. One respondent encapsulated this sentiment by stating the following:

The key issue is undoubtedly the matter of evidence. Less so is data privacy and security, as these are issues that can be adequately addressed.

DiGA without pSVV_3

Nevertheless, a certain level of planning uncertainty was identified in the process, stemming from updates to the DiGA guide or alterations in statements during consultations with the BfArM, as articulated by a manufacturer:

One of the most challenging aspects is the disparity between what is theoretically granted regarding research design and the actual decision practices of the BfArM.

DiGA without pSVV_2

In addition, insights into the procedural aspects revealed that both recruitment and observation periods are critically constrained.

#### Key Aspects of Study Design: Strong Tendency Toward Traditional End Points

Regarding the decision on which positive health care effect a future DiGA will be evaluated for and therefore which end point is used in the study design, significant timing variations are observed. DiGA manufacturers, especially those who began developing their product after the DVG was introduced in 2019, typically determine the positive health care effect early in the development phase, often before initiating the coding process:

Ideally, I make the decision before the development begins, as the entire development process needs to be built around this choice.

DiGA with pSVV_1

In contrast, manufacturers with established products leveraging the new DiGA approval pathway as a market entry point did not have this advantage.

In the selection of the clinical end point, manufacturers strongly lean toward medical benefits:

The medical aspects always take precedence.

DiGA with pSVV_2

Demonstrating a positive health care effect without proof of medical benefit is perceived as challenging in 2 ways. One way is to find the appropriate instrument for evidence generation for proving pSVV, “If I look at the list of pSVV, then I wonder for 80% of the things, how am I supposed to prove that?” (DiGA without pSVV_1), while the other lies in finding an agreement with the BfArM on a study design aiming to prove pSVV, “We have to advise relying on the medical benefit...it is not fundamentally that they (BfArM) reject it (pSVV), but we also experience that they...are not so certain about it” (Consultant_5). In addition, it is perceived that other stakeholders in the system are better equipped to evaluate medical benefits compared to pSVV, which could be relevant for price negotiations:

If you believe you can demonstrate medical benefits, that is probably the better path towards price negotiations because it’s something the system understands and can perhaps evaluate better.

Consultant_6

The perceived challenges conclude in a perception of a stronger acceptance of evidence that substantiates a medical benefit, highlighting a potential current bias toward well-known, medically oriented outcomes.

Manufacturers and consultants choose the end points, if possible, based on the example of other DiGA that are already listed and where validated measurement instruments are available:

[The selection criteria are] essentially hard facts in the sense that it has worked before, and there are validated measurement instruments.

Consultant_3

In addition, both listed DiGA and unlisted health applications prioritize economic considerations:

Ultimately, we paid attention to...economic aspects, like the speed at which the study is conducted.

DiGA without pSVV_2

At present, pivotal guidance in the process emanates from consultations with the BfArM. The influence of the conversations is underscored by the following statement:

So as a manufacturer, you would never do anything where the BfArM advice said otherwise.

DiGA without pSVV_5

#### Reasons for Considering a New Pathway: Additional Evidence

Both, consultants and DiGA manufacturers, currently view the use of pSVV primarily as an opportunity for generating additional evidence alongside medical benefits. This perspective was succinctly expressed by one participant, who mentioned the following:

pSVV is included as a secondary endpoint in such a study. If possible, you confirm it, but you would always prefer to demonstrate medical benefits.

Consultant_3

DiGA manufacturers and consultants have pinpointed 3 substantial benefits associated with the implementation of the possibilities given within the new pSVV pathway. Potential differentiation from providers of similar DiGA was mentioned:

But in the case of [indication X], it could well be that with our DiGA, other DiGA are also approved or provisionally approved. In such instances, I could envision that a pSVV might serve as a consideration for or against a DiGA.

DiGA without pSVV_4

Overall positive impacts on sales were also mentioned:

Distribution is also a crucial task we undertake when engaging with physicians, ensuring a clear delineation of what is effective beyond standard practices. Particularly with DiGA, the argument hinges on the fact that the pSVV dimension precisely provides the benefit.

DiGA without pSVV_3

Moreover, there is an expectation of a positive impact on the official price negotiations occurring later in the process between manufacturers and payers. It is highlighted that “The more evidence you have, the greater the likelihood of maintaining certain price points” (DiGA with pSVV_1) and “Possibly, pSVV can indeed be an enhancing criterion (during the price negotiation process) for the respective product” (Regulatory body_2).

#### Hurdles for Considering a New Pathway: Operational Insecurities

When asked about the reasons why manufacturers tend to not use the new possibilities within the pSVV pathway, many (5/7, 71%) manufacturers and consultants referred to a lack of clarity on operationalizing pSVV as outlined in the DiGA guide, particularly concerning the methods applicable for assessing the impact:

But when I look at them [the pSVV key areas], I wonder with about 80% of them, how am I supposed to prove that? And yes, how do I actually incorporate that into studies?

DiGA without pSVV_1

In addition, there is skepticism among DiGA manufacturers and consultants regarding the economic impact of demonstrating pSVV. One participant succinctly expressed this viewpoint by stating the following:

Purely from the procedural and financial perspective—if you were to ask our boss, who is responsible for finances—there is a clear answer: this is an absolute disaster.

DiGA with pSVV_1

At the same time, introducing and establishing new measurement tools like the use of real-world data is deemed too expensive and risky for DiGA manufacturers:

If you had to essentially innovate methods and also deal with the uncertainty...in the worst-case scenario, you would end up with three studies.... That alone costs you a million, just for these studies. No one is willing to foot that bill. And then, if it goes wrong...

Consultant_3

In addition, manufacturers and consultants observed a general uncertainty within the system regarding the concept of pSVV:

Not that they (BfArM) fundamentally reject it, but we experience that they, how should I say, are not so certain about it.

Consultant_5

Moreover, they observed a general lack of knowledge about pSVV that leads to the need for explanation:

There is a significant need for explanation, actually, among the different stakeholders. Both with patients and with doctors. What is it exactly? Why is it good? What is in it for me?

DiGA with pSVV_1

Consultancies sometimes actively discourage the choice of pSVV in the clinical trial:

If you believe you can demonstrate medical benefits, that is probably the better path towards price negotiations because it’s something the system understands and can perhaps evaluate better.

Consultant_6

#### Relevance Perception: Targeted Innovative Technology Is Important for the Health Care System

Looking at the general perception of pSVV as a concept, stakeholders highlighted the systemic benefits, emphasizing the impact on the overall health care system efficiency and supply effects:

It...makes sense from the overall health care system perspective.

Non-DiGA_2

Patient-centric advantages emerged, revealing the influence of DiGA beyond conventional medical outcomes. A consultant illustrated this by pointing out the positive impact of patient empowerment on intervention success:

Individuals with higher health literacy typically respond more effectively to interventions, as they have a better understanding and feel a greater sense of control.

Consultant_1

DiGA might also address practical challenges like long waiting times, presenting substantial value beyond traditional medical considerations:

But if we now say we would compare ourselves to a traditional intervention...then the pSVV...plays an even greater role. Because when I say, I have something here with a positive effect, and it is more accessible than the therapy, that is already a value where I say, it makes a difference.

DiGA with pSVV_2

However, the unique benefits of DiGA, such as the improvement of patient adherence and reduction of therapy-related efforts, often eluded recognition in conventional benefit assessments:

pSVV is crucial for the system, but the evidence must focus on it. Thus, the usability of the product must be ensured and tailored to that. The evidence must reflect the particular added value of these applications.

DiGA without pSVV_5

Regulatory bodies acknowledged this oversight, emphasizing the need to broaden evaluation criteria to encompass these critical aspects:

DiGA can have medical benefits, but they are not solely limited to medical utility.... And these are points that get lost in traditional benefit assessments.

Regulatory body_1

In addition, the political and collaborative nature of the development process that led to the pSVV concept was highlighted: “We had many stakeholders involved there. On the one hand, we had colleagues from self-administration, but we also had the manufacturers [stretching the collective effort] in understanding potential, addressing challenges, and bridging the gap between traditional assessment methods and innovative logics” (Regulatory body_1).

### Analyzing the pSVV Technology Innovation System: 5 Systemic Problems Identified

#### Actors’ Presence Problem in “Entrepreneurial Activities” and “Market Formation”

Examining the participants presently engaged in the pSVV innovation system reveals a discernible pattern. Only one of the DiGA entrepreneurs within this emergent system has developed a product with a primary emphasis on pSVV. Notably, some manufacturers have established market longevity before the introduction of the DVG and thus pSVV; therefore, orienting their product toward medical benefits. Moreover, some actors are concurrently navigating evidence-generation endeavors while exploring alternative pathways for market access in Germany, such as individual “selective contracts” with statutory health insurance under §140a SGB V (Sozialgesetzbuch 5; a German law that covers all regulation concerning statutory health insurance and therefore also covers DiGA and selective contracts), which lacks the concept of pSVV. The absence of *entrepreneurial activities* singularly centered on pSVV emerges as a contributory factor hindering the *formation of a distinct market* for dedicated pSVV-related regulated digital medical devices.

#### Actors’ Capability Problem in “Knowledge Dissemination”

The supervising governmental institution, in its role as advisor for manufacturers before and during the admission process, has limited experience with pSVV but rather a strong focus on medical benefits. This emphasis can be attributed to its dual role, as the BfArM in Germany oversees admissions not only for DiGAs but also for traditional pharmaceuticals. The lack of experience with pSVV in this key role as a knowledge multiplicator and also in this specific innovation system hampers *knowledge dissemination*. In addition, practitioners prescribing DiGA as well as end users of the products have limited experience with products focusing on pSVV, which leads to a need for significant efforts to explain the concept while the demand from potential users is unclear.

#### Infrastructure Presence Problem in “Knowledge Development” and “Guidance of Search”

The suitability of existing measurement methods designed for therapeutic effects in assessing pSVV is often limited, contributing to its restricted adoption. Simultaneously, the lack of positive examples for products in the pSVV category hampers *knowledge development*. In addition, the absence of positive price examples not only further constrains manufacturers’ support for pSVV but also contradicts the *guidance of search*.

#### Institution Intensity Problem in “Knowledge Development” and “Resources Mobilization”

In the *institutional* dimension, it was noted in particular that the existing *instructions* (DiGA guide) pose challenges to the operationalization of pSVV. The *established practices* and formed *expectations* regarding pSVV are perceived as counterproductive, particularly for cost-sensitive manufacturers who cannot afford to allocate their limited resources toward pSVV despite existing barriers. The limitations of the DiGA guide concerning pSVV, namely lack of examples or standards and possible end points for evidence demonstration, are identified as a challenge in *knowledge development*, while the *established practices*, inclined toward medical benefits, hinder *resource allocation* for the advancement of pSVV.

#### Institution Intensity and Interaction Intensity Problem in “Guidance of the Search,” “Market Formation,” and “Creation of Legitimacy”

*Established practices* in the approval process that make it difficult to enter the market without proven medical benefits diverge from the introduced laws and regulations, creating a misalignment. Interactions within the approval process, especially with the official advisory body, tend to emphasize medical benefits. Notably, consultancies actively guide manufacturers toward prioritizing medical benefits over pSVV, thus steering the guidance of the search away from pSVV and hindering market formation.

Positive expectations on the value of using pSVV that would support the *creation of legitimacy* remain mainly hypothetical. The economic effect, specifically, was formulated in the subjunctive by interviewees. Coupled with the pervasive *established practices*, this poses a significant obstacle to *creating legitimacy* for pSVV.

## Discussion

### Principal Findings

Our research explored the factors that need to be considered when bringing a specific digital health technology innovation to market through the implementation of a regulated market access pathway as a health policy approach.

For this example case study, the pSVV pathway, the descriptive analysis revealed that only a few DiGAs currently in the market have demonstrated a pSVV. Of the 11 currently listed DiGAs that have entered the market with a pSVV as one element of a positive health care effect since the introduction of the pathway in 2019, only 1 DiGA has a primary focus on pSVV (ie, pSVV as the primary end point of the positive health care effect). Notably, 2 of the products (developed by the same DiGA manufacturer) that demonstrate pSVV as a secondary end point conducted their clinical studies before 2019. This indicates that few manufacturers have taken the initiative to develop a product with a special focus on improving structures and procedures for patients, even though the introduction of the pSVV pathway was intended to encourage this. To date, pSVV is mostly used as an additional end point to medical benefits. To provide a basis for interpreting the results in further analyses on the performance factors of innovation systems shaped by regulated market access pathways, our initial conclusion is that the innovation in DiGAs targeting pSVV, which the pSVV pathway was designed to foster, has not yet been fully realized within the system. This is particularly daunting, as previous studies emphasize the need to focus more on products that address structural and procedural improvements for patients [53-55] and consider the concept of pSVV to be a “fundamental mechanism of action for DiGAs” [56].

Our qualitative analysis of stakeholders’ perspectives during market access reveals that evidence generation is the most critical component of the DiGA approval process. While multiple factors are considered in determining a positive health care effect, our findings suggest that, at the operational level, manufacturers of DiGAs primarily focus on demonstrating medical benefits as evidence for positive health care outcomes (Figure 2). Economic considerations, in light of the lack of established methodologies for measuring the defined key areas and uncertainty about the acceptability of the proposed study design, including pSVV, lead to more cautious use. Bearing the cost for methodological innovation is not considered attractive among manufacturers, particularly as DiGAs with pSVV so far do not negotiate substantially higher prices than DiGAs without pSVV [28]. Consistent with the findings of the descriptive analysis, the use of pSVV is often considered “incidentally” as an addition to medical benefit, only when a methodology for demonstrating one of the pSVV key areas exists for a specific product (Figure 3). It can be concluded that the specific challenges associated with proving the efficacy of pSVV, particularly the lack of established methodologies, currently outweigh its potential benefits. Previous studies have highlighted content and format security [57] as a critical factor, particularly for pioneers in medical technology, which also appears to hold true for the market entry of innovative products in DiGAs. The fact that only one company has attempted to pioneer a product specifically targeting benefits under pSVV may be attributed to the innovators’ need for content and format security, in this case specifically the lack of standards to generate evidence for the targeted technology innovation. Without such security, the costs for pioneers become prohibitively high.

However, the implementation of the pSVV concept is recognized as vital for the health care system’s evolution itself (Figure 4).

**Figure 5:**
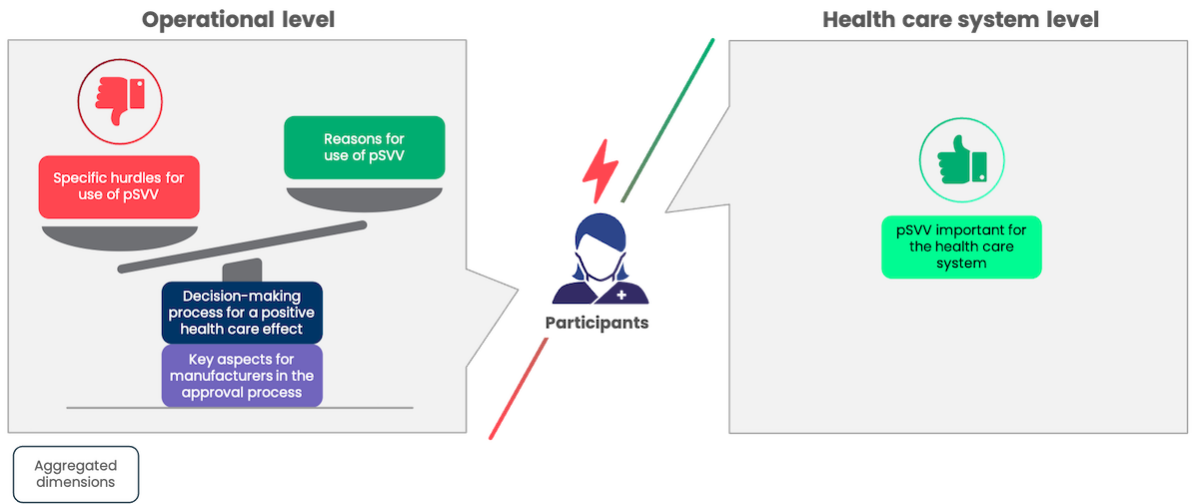
Underlying conflict leading to the underuse of patient-relevant structural and procedural improvements (pSVVs).

Our qualitative analysis highlights a common gap in health care innovation between the perceived importance of new technologies and their actual implementation. This gap is often influenced by systemic factors, such as regulatory conditions and inherent system inertia, alongside a general openness to innovation among stakeholders [58]. For the pSVV pathway, while there is strong stakeholder support for introducing products that demonstrate pSVV, significant operational hurdles, particularly concerning content and format security, have delayed its adoption. These barriers suggest that, despite a willingness to embrace this innovation, the pathway’s practical challenges currently limit its broader impact (Figure 5).

To further explore performance factors in the innovation system shaped by the pSVV pathway, we conducted an analysis of the innovation system’s maturity level in relation to pSVV. Our findings indicate 3 central strategic factors of an innovation system in which we can expedite the adoption of innovative technology: *framework specification*, *active innovation process* *management,* and *market formation stimulation*

While previous work has highlighted the general need for frameworks [16,59], our emphasis is on the degree of specificity required for the successful implementation of a market access pathway for DiGAs. In our example, the specification of the pSVV pathway, defined in the DiGA guide, requires 2D adjustments. First, the creation of a knowledge infrastructure would enable entrepreneurs to navigate the DiGA market, including pSVV, better if the development of exemplary models for pSVV products and especially congruent methodologies for demonstrating the evidence base of pSVV would exist. In addition, a multitude of studies highlight the ongoing challenges associated with identifying appropriate study designs that are capable of measuring effects solely attributable to the use of digital products [60-62]. We conclude that the presence of a specific level of knowledge infrastructure, which underpins the innovation driven by the introduction of a regulated market access pathway, can affect how widely it is adopted [6,63].

Second, studies in the field of regulated medical products have indicated that the uncertainty about the accepted content and format during the approval of new products can make the pathway into the market unattractive for first movers [57]. Our analysis has shown that the DiGA guide, which serves as the most relevant instruction at the operational level in this system, requires timely updates to keep pace with advancing knowledge development and ensuring up-to-date format and content security. Simultaneously, for the successful implementation of a pathway, *active innovation process management* is crucial. Providing training to key actors in the defined process, such as the BfArM or consultants, has been shown to be essential for empowering stakeholders to support and advance pSVV as an innovation. In addition, as previous work has highlighted the importance of education and awareness as well as patient and health care provider support while training for physicians on digital health solutions is limited [25,64], the inclusion of user groups and prescribing physicians can be focused to sharpen the objectives of pSVV. Conversely, it is crucial to ensure that involved actors are not overly familiar with traditional processes, as this can lead to inflexible adherence to these methods. The involvement of key actors with an excess of experience in medical benefits (ie, experienced regulatory assessor previously involved in assessing pharmaceutics) could potentially impede innovation speed if their participation is overly predominant.

Furthermore, targeted *market formation stimulation* can aim to introduce manufacturers into the DiGA market, focusing primarily on addressing pSVV key areas [65]. Creating positive examples can accelerate innovation at multiple levels. In addition, stimulating constructive exchange regarding the pSVV concept can expedite knowledge generation and distribution, contributing to faster market formation.

In summary, our study revealed that the lack of established methodologies for proving pSVV has led to significant uncertainty for manufacturers regarding the acceptable formats and evidence required, contributing to slower adoption. To complement these findings on the specific pathway level, the functional-structural analysis identified 3 key factors critical to enhancing the total innovation system: clearer framework specification to provide precise guidelines, active innovation process management to support stakeholders in navigating the regulatory process, and market formation stimulation to foster positive examples and increase engagement with the pSVV pathway. These factors are essential for overcoming both the operational and systemic barriers to successfully introducing innovative digital health technologies into a market.

As initiators of the systemic innovation of pSVV, the regulatory body can implement additional measures to promote its further establishment. This includes stimulating the development of a knowledge infrastructure by involving all stakeholders in the innovation system. Academia can actively contribute to advancing the definition of the pSVV and particularly developing suitable methodologies for evidence generation [66-68]. Existing mechanisms to promote scientific progress, such as dedicated research funds (eg, in Germany by the Federal Ministry of Education and Research or the Innovation Fund of the Federal Joint Committee), could be set up for this objective [69].

Initiating a public debate on pSVV can engage relevant stakeholders, such as professional societies and associations in a discourse to further develop the pSVV framework [13,66]. Economic measures, such as granting financial support for conducting clinical trials assessing pSVV or even tax incentives for implementation, can provide additional incentives for manufacturers driving innovation and initiating the pSVV market until these products are established in the standard of care. Furthermore, spaces can be created for key stakeholders in the system to educate themselves about pSVV. This could include offering policy laboratories or training programs for knowledge multipliers.

Consultancies and the BfArM, as influential players in the system, can provide room for their employees to further educate themselves about the possibilities of pSVV, to prevent disproportionately biased inclinations toward medical benefits. Ensuring a balanced wealth of experience regarding the use of medical benefits within the workforce can promote openness to innovative processes. It is critical to ensure that the experience of the staff responsible for evaluating the proof of benefit of DiGAs is balanced in assessing medical quality [70]. Previous work has shown that the Food and Drug Administration might actively aid in similar innovation processes [71]. This assistance is rendered through the administration’s in-depth knowledge to improve study designs and by championing innovative methods. Although the breadth of authority and scope of the regulated processes vary, BfArM or other regulatory agencies involved in the approval process and specifically in evaluating coverage eligibility can also contribute their expertise. For manufacturers seeking to use the potential of pSVV in their DiGAs, it is important to prioritize evidence of positive health care effects early in the development process. Identifying the “active ingredient” and its mechanism is crucial for crafting studies effectively assessing DiGA [60].

**Figure 6:**
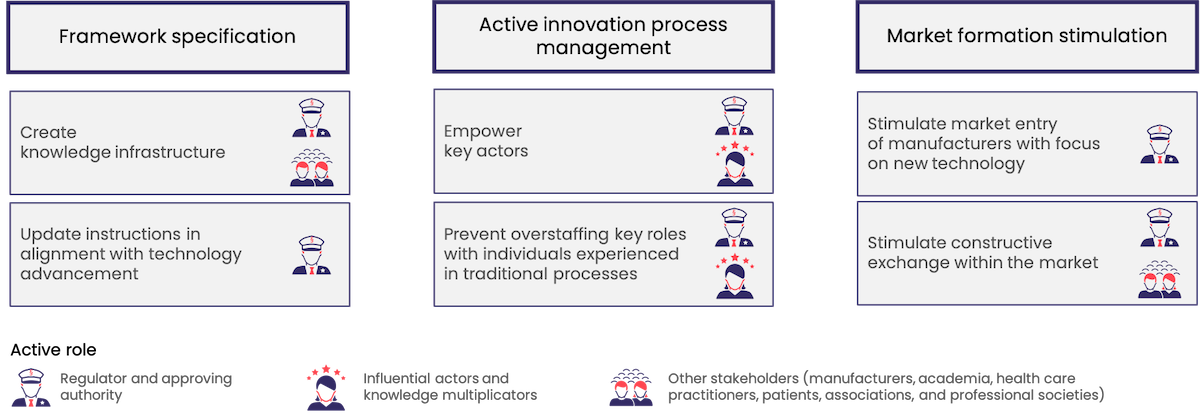
Central strategic factors for progress exploration in the innovation system derived from an exemplary analysis of the patient-relevant structural and procedural improvement pathway.

Besides proposing solutions for individual stakeholder groups within the health care system (Figure 6), the collaboration and coordination among all involved parties should be underscored as crucial for success. As highlighted on an international scale, effective collaboration between regulatory agencies, academia, app developers, payers, and health care providers is essential to truly leverage advances in science and technology and to translate the vision into reality, guided by the right incentives and guidelines [69,71,72].

Our analysis suggests that the strategic factors identified are valuable considerations for the establishment of a regulatory market access pathway, to ensure its widespread and successful adoption. Drawing on previous research, the strategic factor of content and format security [57] is highlighted, alongside the adoption of recognized and accepted methods for measuring proposed parameters [59,61,68,73-76]. This is particularly relevant in digital health technology aimed at patient empowerment, where significant progress is still needed [68,74]. Furthermore, it is advantageous to strike an optimal balance between leveraging established processes and frameworks allowing room for innovation [77]. As indicated by the strategic factor of innovation process management, it is crucial to carefully select and train key players and individuals in pivotal roles, equipping them with the specific skills and mindset needed to drive innovation. Finally, the strategic factor of market formation stimulation points to the critical need to consider the practicality of a framework in enabling a viable business model, particularly within the context of systemic innovation [21,60,78].

### Limitations

Acknowledging the limitations of this study, a conclusive assessment of the economic value of pSVV is precluded, as no product with pSVV as a primary end point has undergone final price negotiation yet (Table S2 in Multimedia Appendix 1). In addition, it could be questioned whether pSVV can ever be the primary end point of a DiGA, as all DiGAs must also be CE–certified medical devices with the corresponding intended purpose. We have not explored this discussion in detail, as it can certainly be conducted more effectively with selected examples of products focusing on pSVV, once these are available in the market. Furthermore, our qualitative analysis, conducted through semistructured interviews, did not encompass all functional-structural dimensions of innovation systems. Entities, such as academia or associations were not mentioned by any interviewees and were thus not part of the evaluation. In addition, our sampling strategy did not include patients or physicians, as the study concentrated on identifying reasons for the scarce number of products targeting the pSVV key focus areas. This decision acknowledges the limited familiarity of end users and prescribers with the pSVV concept at this point and their marginal impact on market entry decisions shortly after the introduction of a new framework designed to facilitate market access.

### Conclusions

This exploratory study examined critical factors for bringing digital health technology innovation to market through a regulated market access pathway, with a focus on the pSVV pathway as an example case. Key success factors identified included content and format security, the need for precise framework specification, active innovation process management, and market formation stimulation. These elements collectively help reduce uncertainties for manufacturers and promote broader adoption of digital health innovations. Our findings revealed that although the pSVV pathway was intended to encourage a focus on pSVVs, the current use remains limited, with most manufacturers prioritizing medical benefits as evidence of positive health care outcomes.

The mixed methods approach enriched the analysis by allowing a deep exploration of stakeholder perspectives through qualitative analysis, complemented by the functional-structural analysis of the technology innovation system. The combination of grounded theory and Gioia methodology enabled an objective and detailed examination of how market participants view and use the pSVV pathway, while the systemic analysis highlighted additional success factors in the broader innovation system context.

Our findings contribute valuable insights for stakeholders in the digital health sector, providing recommendations for policy adjustments that can strengthen innovation pathways. With this work, we contribute by identifying critical areas for improvement, including the need for clearer guidelines, the development of a knowledge infrastructure, and enhanced training for stakeholders to help overcome operational barriers. In addition, fostering public discourse on pSVV and introducing economic incentives, such as financial support for clinical trials, may further drive innovation. These recommendations are valuable for health care systems and regulatory bodies seeking to support similar pathways and advance patient-centered digital health solutions.

Furthermore, the impartial perspectives gathered from stakeholders, combined with a holistic analysis of the innovation system, contribute to identifying clear fields for action and proposing solutions relevant beyond this specific case. Our findings provide transferable insights for health care systems embarking on similar systemic innovations, as seen in countries like France, Belgium, and Austria, where comparable concepts to pSVV are being introduced. The implications of our research extend to other systemic innovation initiatives in health care systems facing significant adoption challenges that could otherwise lead to initiative failure. Ongoing efforts from national and international scholars are critical in supporting the success of these initiatives. In Germany, for example, a reimbursement pathway for digital nursing care applications (DiPA), a “regulatory sibling” to DiGA, has been established. While speculative, similar challenges may emerge for DiPA manufacturers, particularly concerning the nursing benefit that aligns with both medical benefit and pSVV, possibly contributing to the current absence of DiPA in the marketplace and highlighting gaps in this systemic innovation approach.

## Appendix

Multimedia Appendix 1 Semistructured interview questionnaires, overview of interview participants, full data structure based on Gioia methodology, negotiated digital health application prices and health care effects, selected interview codes generated using grounded theory, functional-structural analysis of patient-relevant structural and procedural improvement as a systemic innovation, and derivation of strategic fields using the functional-structural framework by Wieczorek and Hekkert [34].

## Abbreviations

BfArM: Federal Institute for Drugs and Medical Devices (German: Bundesinstitut für Arzneimittel und Medizinprodukte)
CE: Conformité Européenne
COREQ-32: Consolidated Criteria for Reporting Qualitative Research-32
DiGA: digital health application (German: Digitale Gesundheitsanwendung)
DiGAV: digital health application ordinance (German: Digitiale Gesundheitsanwendungen-Verordnung)
DiPA: digital nursing care application
DVG: Digital Healthcare Act
PECAN: Prise en Charge Anticipée Numérique
pSVV: patient-relevant structural and procedural improvement (German: Patientenrelevante Struktur- und Verfahrensverbesserung)

## Data Availability

The datasets generated and analyzed during this study are not publicly available due to data protection reasons but are available from the corresponding author on reasonable request.
