# Supplementary material for: Unpacking Performance Factors of Innovation Systems and Studying Germany’s Attempt to Foster the Role of the Patient Through a Market Access Pathway for Digital Health Applications (DiGAs): Exploratory Mixed Methods Study
Source: J Med Internet Res. 2025 Jan 6;27:e66356. doi: 10.2196/66356 (PMC11747537; doi:10.2196/66356)
Supplement: Multimedia Appendix 1 [file jmir_v27i1e66356_app1.docx]

Supplementary Information

**Unpacking Performance Factors of Innovation Systems: An exploratory mixed-methods study of Germany's attempt to foster the role of the patient through a market access pathway for Digital Health Applications (DiGAs)**

Table of contents

[List of illustrations 2](#_Toc164427050)

[List of tables 2](#_Toc164427051)

[Supplementary methods 3](#_Toc164427052)

[Questionnaire semi-structured interview part A (Manufacturer of Regulated Medical Devices; listed as DiGA) 3](#_Toc164427053)

[Questionnaire semi-structured interview part B (Manufacturer of Regulated Medical Devices; Non-DiGA) 4](#_Toc164427054)

[Questionnaire semi-structured interview part C (Experts in the field of Digital Health Applications) 5](#_Toc164427055)

[Supplementary information research sample: Overview interview participants 6](#_Toc164427056)

[Supplementary figures 7](#_Toc164427057)

[Data Structure based on Gioia Methodology 7](#_Toc164427058)

[Supplementary tables 8](#_Toc164427059)

[Negotiated DiGA prices and the underlying positive healthcare effects 8](#_Toc164427060)

[Selected Codes generated from Interviews Using Grounded Theory 9](#_Toc164427061)

[Functional-Structural Analysis of pSVV as a Systemic Innovation from Concepts generated in Qualitative Analysis 21](#_Toc164427062)

[Derivation of Strategic Fields from Systemic Problems Using the Functional-Structural Framework of Wieczorek & Hekkert 30](#_Toc164427063)

[Glossary 32](#_Toc164427064)

[Additional remarks 32](#_Toc164427065)

[Bibliography 32](#_Toc164427066)

# List of illustrations

[Figure 1 Data Structure based on Gioia Methodology 7](#_Toc164193392)

# List of tables

[Table 1 - Supplementary information interview participants 6](#_Toc164427040)

[Table 2 - List of negotiated prices for all DiGA that have already undergone the official negotiation process (as of January 31,2024)^1^ 8](#_Toc164427041)

[Table 3 - Selected codes generated from interviews using grounded theory 20](#_Toc164427042)

[Table 4 - Functional-structural analysis of pSVV as a systemic innovation from concepts generated from theme (2)-(5) in the qualitative analysis 29](#_Toc164427043)

[Table 5 – Derivation of strategic instruments to address identified systemic problems in alignment with functional-structural analysis framework^2^, clustered into actionable strategic fields 31](#_Toc164427044)

# Supplementary methods

## Questionnaire semi-structured interview part A (Manufacturer of Regulated Medical Devices; listed as DiGA)

**Fragestellung Anbieter von digitalen Gesundheitsanwendungen
(gelistet im BfArM DiGA Verzeichnis) – Original German
Zulassungsprozess allgemein**

- Was sind für euch die Schlüsselthemen im Zulassungsprozess?
  - Vorläufiger Zulassungsprozess
  - Dauerhafter Zulassungsprozess
- Wie und wann wurde entschieden, welcher positive Versorgungseffekt genutzt werden soll?
- Habt ihr noch andere positive Versorgungseffekte (z.B. pSVV) in Betracht gezogen?
- Wer hat das Design der entsprechenden Studie konzipiert und wieso habt ihr euch für das gewählte Design entschieden?

**Nachweis einer patientenrelevanten Struktur- und Versorgungsverbesserung (pSVV)**

- Welche Relevanz hat für euch die Möglichkeit eine pSVV nachzuweisen, um einen positiven Versorgungseffekt nachzuweisen?
- Welche Chancen seht ihr in dieser Möglichkeit, die das BfArM zusätzlich zum Nachweis des medizinischen Nutzens bietet?
- Wo liegen aus eurer Sicht die größten Herausforderungen speziell für den Nachweis von pSVV?

**Research Inquiry for Providers of Digital Health Applications (Listed in the BfArM DiGA Directory) – Translation English
Admission process overview**

- What are the key themes in your admission process?
  - Preliminary listing considerations
  - Permanent listing considerations
- How and when was the decision made regarding the utilization of a specific positive care effect?
- Were other positive care effects (e.g., pSVV) considered?
- Who conceptualized the design of the corresponding study, and why was the chosen design preferred?

**Demonstration of patient-relevant structural and procedural improvement (pSVV)**

- How significant is the ability to demonstrate a pSVV for establishing a positive care effect?
- What opportunities do you perceive in this capability, which the BfArM provides in addition to demonstrating medical benefit?
- From your perspective, what are the greatest challenges, specifically in proving pSVV?

## Questionnaire semi-structured interview part B (Manufacturer of Regulated Medical Devices; Non-DiGA)

**Fragestellung Anbieter von digitalen Gesundheitsanwendungen (nicht gelistet im BfArM DiGA Verzeichnis) – Original German
Zulassungsprozess allgemein**

- Wie und wann habt ihr entschieden, dass ihr euch nicht als DiGA im BfArM Verzeichnis listen lassen wollt?
- Welche Gründe haben zu dieser Entscheidung geführt?
- Was sind für euch die Schlüsselthemen im BfArM Zulassungsprozess, die dazu geführt haben, dass ihr euch entschieden habt, diesen nicht zu durchlaufen?
- Falls zutreffend: Welche positiven Versorgungseffekte hattet ihr diskutiert?

**Nachweis einer patientenrelevanten Struktur- und Versorgungsverbesserung (pSVV)**

- Wie blickt ihr auf die Relevanz der Möglichkeit eine pSVV nachzuweisen, um einen positiven Versorgungseffekt nachzuweisen?
- Welche Chancen seht ihr in dieser Möglichkeit, die das BfArM zusätzlich zum Nachweis des medizinischen Nutzens bietet?
- Wurde für euer Produkt/eines eurer Produkte der Nachweis einer pSVV erwogen?

**Research Inquiry for Providers of Digital Health Applications (not listed in the BfArM DiGA Directory) – Translation English
Admission process overview**

- How and when did you decide not to list your digital health application as a DiGA in the BfArM Directory?
- What reasons led to this decision?
- What are the key aspects of the BfArM admission process that influenced your choice not to undergo it?
- If applicable, what positive care effects were discussed?

**Demonstration of patient-relevant structural and procedural improvement (pSVV)**

- From your perspective, how do you perceive the relevance of demonstrating a pSVV to establish a positive therapeutic effect?
- What opportunities do you see in this approach, provided by the BfArM in addition to proving medical benefit?
- Was the demonstration of a pSVV considered for your product/any of your products?

## Questionnaire semi-structured interview part C (Experts in the field of Digital Health Applications)

**Fragestellung Experten im Bereich digitale Gesundheitsanwendungen – Original German
Zulassungsprozess allgemein**

- Was sind aus deiner Sicht die Schlüsselthemen im Zulassungsprozess?
  - Vorläufiger Zulassungsprozess
  - Dauerhafter Zulassungsprozess
- Wie und wann wird entschieden, welcher positive Versorgungseffekt genutzt werden soll?
- Wer konzipiert das Design der entsprechenden Studie?
- Welche Designs werden deiner Erfahrung nach am häufigsten verwendet?

**Nachweis einer patientenrelevanten Struktur- und Versorgungsverbesserung (pSVV)**

- Welche Relevanz hat für euch die Möglichkeit eine pSVV nachzuweisen, um einen positiven Versorgungseffekt nachzuweisen?
- Welche Chancen seht ihr in dieser Möglichkeit, die das BfArM zusätzlich zum Nachweis des medizinischen Nutzens bietet?
- Gibt es pSVV, die „leichter“ nachzuweisen sind?

**Research Inquiry for Experts in the Field of Digital Health Applications – Translation English
Admission process overview**

- What are the key themes in your admission process?
  - Process for preliminary listing
  - Process for permanent listing
- How and when is the decision made regarding which positive care effect to leverage?
- Who designs the study corresponding to this effect?
- In your experience, what are the most commonly utilized research designs?

**Demonstration of patient-relevant structural and procedural improvement (pSVV)**

- How significant is the ability to demonstrate a pSVV for establishing a positive care effect?
- What opportunities do you see in this capability, which the BfArM offers in addition to proving medical benefit?
- Are there pSVV that are comparatively "easier" to demonstrate?

# Supplementary figures

## Data Structure based on Gioia Methodology


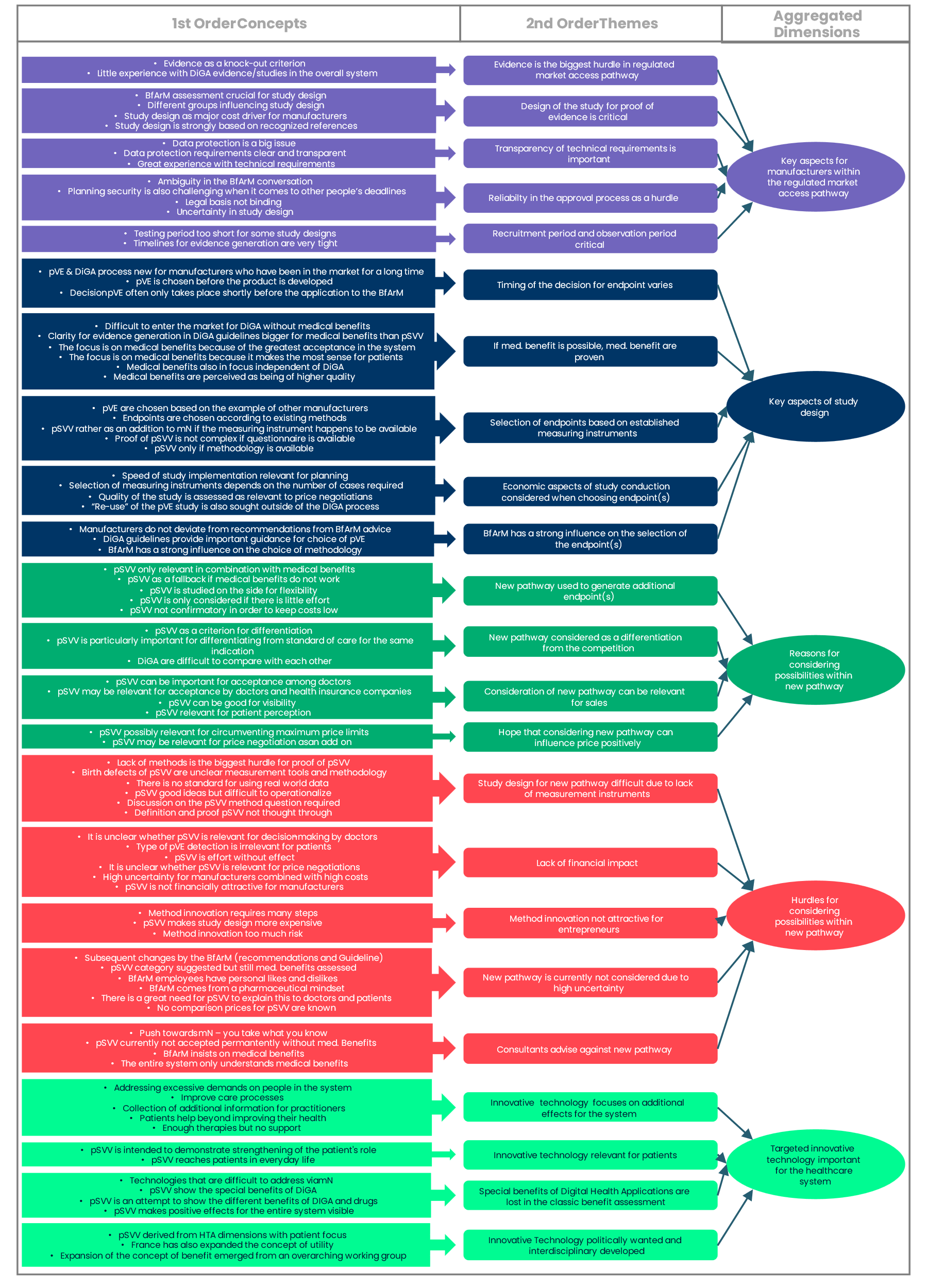


Figure 1 Data Structure based on Gioia Methodology

# Supplementary tables

## Supplementary information research sample: Overview interview participants

| **#** | **Stakeholder group** | **Professional position** | **Gender** |
| --- | --- | --- | --- |
| 1 | Consultants | Managing Partner | Male |
| 2 | Consultants | Senior Consultant | Male |
| 3 | Consultants | Senior Consultant | Female |
| 4 | Consultants | Managing Partner | Male |
| 5 | Consultants | Senior Consultant | Male |
| 6 | Consultants | Scientific Director | Male |
| 7 | DiGA with pSVV | Science Lead | Female |
| 8 | DiGA with pSVV | Head of Market Access | Male |
| 9 | DiGA without pSVV | CEO | Male |
| 10 | DiGA without pSVV | Science Lead | Female |
| 11 | DiGA without pSVV | CEO | Male |
| 12 | DiGA without pSVV | Chief of Staff | Male |
| 13 | DiGA without pSVV | Founder | Male |
| 14 | Digital Medical Products (Non-DiGA) | CEO | Male |
| 15 | Digital Medical Products (Non-DiGA) | CEO | Male |
| 16 | Digital Medical Products (Non-DiGA) | Medical Director | Female |
| 17 | Regulatory Body | Other leadership position | Male |
| 18 | Regulatory Body | Other leadership position | Male |
| 19 | Regulatory Body | Other leadership position | Male |

Table 1 - Supplementary information interview participants

## Negotiated DiGA prices and the underlying positive healthcare effects

| **DiGA** | **Positive Healthcare Effect** | **Price per 3 months**  **(after official negotiation process)** |
| --- | --- | --- |
| Cara Care für Reizdarm | Medical Benefit + pSVV | 248,00 € |
| Kranus Edera | Medical Benefit + pSVV | 235,00 € |
| Mindable: Panikstörung und Agoraphobie | Medical Benefit + pSVV | 245,50 € |
| Velibra | Medical Benefit + pSVV | 230,00 € |
| Vorvida | Medical Benefit + pSVV | 192,01 € |
| companion patella powered by medi - proved by Dt. Kniegesellschaft | Medical Benefit | 223,49 € |
| Deprexis | Medical Benefit | 243,00 € |
| Edupression.com | Medical Benefit | 224,80 € |
| Elevida | Medical Benefit | 243,00 € |
| HelloBetter Diabetes und Depression | Medical Benefit | 222,99 € |
| HelloBetter Panik | Medical Benefit | 230,00 € |
| HelloBetter ratiopharm chronischer Schmerz | Medical Benefit | 235,00 € |
| HelloBetter Stress und Burnout | Medical Benefit | 235,00 € |
| HelloBetter Vaginismus Plus | Medical Benefit | 235,00 € |
| Invirto- Die Therapie gegen Angst | Medical Benefit | 220,00 € |
| Kaia Rückenschmerzen | Medical Benefit | 221,49 € |
| Kalmeda | Medical Benefit | 189,00 € |
| Neolexon Aphasie | Medical Benefit | 223,01 € |
| NichtraucherHelden-App | Medical Benefit | 211,00 € |
| Novego: Depressionen bewältigen | Medical Benefit | 199,00 € |
| Oviva Direkt für Adipositas | Medical Benefit | 220,90 € |
| PINK! | Medical Benefit | 234,50 € |
| Selfapys Online-Kurs bei Binge-Eating-Störung | Medical Benefit | 232,00 € |
| Selfapys Online-Kurs bei Bulimia Nervosa | Medical Benefit | 232,00 € |
| Selfapys Online-Kurs bei Depression | Medical Benefit | 217,18 € |
| Selfapys Online-Kurs bei Generalisierter Angststörung | Medical Benefit | 228,50 € |
| Somnio | Medical Benefit | 224,99 € |
| Vivira | Medical Benefit | 206,79 € |
| zanadio | Medical Benefit | 218,00 € |

Table 2 - List of negotiated prices for all DiGA that have already undergone the official negotiation process (as of July 1st,2024)^1^

Kindly note that prices are presented for 29 Digital Health Applications (DiGA), notwithstanding the fact that a total of 35 DiGA are currently permanently listed. This discrepancy is attributed to a temporal delay existing between the permanent listing and the culmination of official price negotiation procedures.

A Mann-Whitney U test was conducted to examine the correlation between the pricing and the type of positive healthcare effect utilized. At a significance level of Alpha = 0.05, no significant difference in prices is evident between the utilization of "medical benefit" and "medical benefit + pSVV."

## Selected Codes generated from Interviews Using Grounded Theory

| **Theme** | **Theme Description** | **Selected Quotes** |
| --- | --- | --- |
| **Dimension 1: Key aspects for manufacturers within the regulated market access pathway** | | |
| Evidence is the biggest hurdle in regulated market access pathways | The predominant factor consuming significant time and contributing to uncertainty in the regulatory approval process, most frequently resulting in non-inclusion in the DiGA directory, is the aspect of evidence. | "And I believe the biggest issues that have concerned us were indeed uncertainty regarding the evidence that is generated. So, what evidence do we need to generate? What are the requirements here? Will the endpoints be accepted in the given form? These are the issues that, I believe, have strongly preoccupied people. And also, I would say, have brought a lot of uncertainty into the whole thing. Because it simply wasn't entirely clear - will it be perceived as positively as we have done, even if, of course, there has been consultation. There are very, very few guidelines on the topic out there. It's quite different, I would say, from classical pharmaceutical market access, where it's already very established, or there are actually clear guidelines on how such evidence should look and how it is then evaluated. And all of that doesn't exist here." (DiGA with pSVV_2)  "The key issue is undoubtedly the matter of evidence. Less so is data privacy and security, as these are issues that can be adequately addressed." (DiGA without pSVV_3)  "So, the most critical point is, of course, the evidence, the study situation - point. I believe everything related to that. I have the feeling, and this is also what manufacturers most frequently have problems with or what is often the crucial point, which often decides on inclusion or non-inclusion." (Consultant_5)  "Otherwise, of course, I closely follow from conversations with other DiGA manufacturers what their issues are. But I still always hear, above all, it is evidence, evidence, evidence." (Health app_2) |
| Design of the study for proof of evidence is critical | This theme encapsulates the significance of meticulous study design. Key codes reveal the pivotal role of BfArM assessment in shaping design, the diverse influencers impacting decisions, manufacturers viewing it as a major cost factor, and the foundation of study design on established references, highlighting its criticality in generating robust evidence. | "So, one has become more precise, actually one would say, one has also become more precise. Especially in the protocols one writes, it has to be very clear how you analyze the data in the end. You can, so the statistical analysis plan must be formulated quite clearly before the start of the study. So, the pre-specification is mega important. It's something where maybe the scientific world doesn't always look so closely. And then you make a small change in the analysis procedure or in the study, for whatever reasons, but that is no longer possible with the BfArM. They attach great importance to you having everything really clear before you start reporting." (DiGA without pSVV_3)  "So, of course, in theory, it is possible to build other prospective comparative studies as well. But well, if the quality of evidence is lower, the chance that the BfArM accepts the study also decreases. Yes, and so far, only RCTs have been accepted." (Consultant_5)  "And one can clearly see that an evidence level is crystallizing, which, within the framework that is possible, is already on the strictest side. So far, besides RCTs, there is no other evidence for final approval." (DiGA without pSVV_4)  "There are simply very, very few guidelines on the topic out there. It's quite different, I would say, from classical pharmaceutical market access, where it's already very established, or there are actually clear guidelines on how such evidence should look and how it is then evaluated. And all of that doesn't exist here. That, of course, has its advantages and disadvantages, one must say honestly. So, advantages may also be that if something is not quite clearly defined, there may be the possibility to discuss it and then it might be accepted after all, the concept. And the disadvantage is, you're just sitting there and not quite sure, until the end, will this be enough or not." (DiGA with pSVV_2)  "In terms of evidence, the question always arises about how the systematic data evaluations should actually look. How many patients should be included is always very important because that is very unclear. If we're talking about 40, or over 60, that's already a big difference. And that is also a major cost driver. If you then have to do a small preliminary study in the same study, it is very cost-intensive." (DiGA without pSVV_3) |
| Transpa-rency of technical require-ments is important | This theme underscores the dual nature of navigating technical demands in qualitative analysis. While data protection poses a substantial challenge, the clarity and transparency of the associated requirements alleviate concerns. Participants express a positive sentiment, highlighting a commendable experience in managing the intricate technical aspects of the research process. | "So, these tech matters and such, it's indeed a huge stress in the company, but I can understand all of that - we all just have to work on weekends, day and night, and that works quite well. But a study design, suddenly setting up a control arm, well, there I simply have no chance anymore." (DiGA with pSVV_1)  "The next effect, where most drop out, is then, of course, data protection-information security. It's not actually thinking once about what I have to do for the product, I now have to do cyber-security, what are the requirements, how the whole thing is structured, and what do I have to do as a company. That means, DiGA also demands company certifications [before ISMS, so after 2701, there is a data protection certification there through this catalog from BSE, BMG, and BFARM, these stories, that is far from the product. And there, people in these companies didn't think enough about what has to be done to classify this in this timeline. But especially in the area of study planning. An effect is opened up where you think, yes, that lowers blood pressure. And then I fail at DiGA later because suddenly I need a study with 1000 female subjects. And that's a problem. You have to know that beforehand." (Consultant_3)  "But still, I always hear, above all, it's evidence, evidence, evidence. That doesn't mean there aren't other issues, right? I mean, sure, there's also the issue of data protection. I'm curious about what's coming up soon when all these interoperability standards somehow have to be included, which issues will suddenly be on the agenda. Then there's the big question, well, is implementing any interoperability interfaces a significant change?" (Health app_2) |
| Reliability in the approval process as a hurdle | This theme exposes challenges within the BfArM conversation. Ambiguity prevails, hindering planning security concerning external deadlines. The perceived non-binding nature of the legal basis adds to the complexity. Uncertainties in study design compound the hurdle, emphasizing the critical need for enhanced reliability throughout the approval process. | "You naturally run the risk as a manufacturer that if the BfArM is not satisfied with the interim analysis or this study design, the patients are completely burned again. You have to start all over again." (DiGA with pSVV_1)  "So, the BfArM would actually be consulted. Well, we asked them, like how do you assess it? We would suggest this to ourselves. And I said, well, I mean, I wonder how much you can rely on what the BfArM says... I mean, it also has a half-life. But in the end, that was agreed with the BfArM. We also have it in writing, but well, that doesn't count for anything." (Health app_2)  "It's a bit problematic that what the BfArM says in the advisory conversation has no real binding effect and is sometimes seen differently in the actual listing process. And this discrepancy is, of course, well, that's really awkward. So, that's really something the BfArM doesn't want, but still, it produces these cases." (Consultant_2)  "One of the most challenging things aspects is the disparity between what is theoretically granted regarding research design and the actual decision practices of the BfArM. They want RCT. Everything else that is possible in the guidelines - like SMART designs, intra-individual comparisons - you can't get that through anyway. And there is, I think, it's important to note again that there is simply a big divergence between what is formally allowed and what is practically desired, which is then something else." (DiGA without pSVV_2) |
| Recruitment period and observation period critical | This theme reveals challenges: the testing period is insufficient for certain study designs, demanding careful reconsideration. Tight timelines for evidence generation add complexity to managing recruitment and observation periods in qualitative analysis. | "It depends, currently, we usually have an average observation time of three months for the intervention, and people cross over, so six months per patient. If I also have the recruitment window, then I won't fit into the time. So, three months is already quite tight. We also have many with six months of observation time, which also makes sense. And then it's simply not possible to squeeze it into 12 months. Especially for anyone who knows well how long recruitment can take. And that is simply not possible." (Consultant_4)  "So, you have to achieve significance in these subgroups. It's more difficult when you basically need 30 times as many people to get it done in these groups. Exactly, you have to power it accordingly, and I only have one year for the study. Well, I can extend it, then I have a maximum of two years, but I see how it's going with us now. It's really tough. So, depending on the indication, you go in and then it's said, well, I understand that too - rare indications simply need fewer cases, that's completely okay - but assuming 'Does it have a license, it will be no problem to get study patients' and we were automatically at 500 patients. That means our task will be to include 500 patients, and that is actually already... well, you need eight a day - which is not feasible for a clinical day. Even if I do it multicentrically, which we will, but it's tough." (DiGA with pSVV_1) |
| **Dimension 2: Key aspects for study design** | | |
| If med. benefit is possible, med. benefit are proven | This theme explores the temporal dynamics in decisions for positive healthcare effects. Manufacturers new to pVE and DiGA face a learning curve. Choosing pVE before development contrasts with decisions often made shortly before BfArM application, revealing variations in the timing of impactful healthcare decisions. | "Well, unfortunately, we have to advise relying on the medical benefit somehow. [...] No, with this construct of positive structural and procedural improvement, patient-relevant structural and procedural improvement - not fundamentally that they (BfArM) reject it, but we also experience that they, how should I say, are not so certain about it. So, they find it difficult." (Consultant_5)  "The medical aspects always take precedence. So, yes, one could say theoretically, one could also have only a positive care effect without proving anything medical. I mean not a positive care effect, I mean patient-relevant structural and procedural improvement, without having an add-on here, but our therapies are developed in such a way that we always have a medical benefit included because we believe that this is how we find the greatest significance and also the greatest acceptance both for the patient and in the system." (DiGA with pSVV_2)  "Yes, so I believe, I already said it earlier, now with the Digital Health Act and the regulations for the higher risk classes, one is trying to take that into account to some extent, that a medical benefit is somehow what is qualitatively more valuable and that is of particular relevance for the statutory health insurance." (Regulatory Body_3)  "We collect everything here and then we make it a bit dependent. We only have to prove one, but the big goal is, of course, the improvement of health status." (Health App_3)  "Our product was actually always designed as a product to clearly address the medical benefit. For us, it was always about creating a therapy, and therapy is something that has a therapeutic effect, so it must provide a medical benefit. That's why it wasn't really an issue, and because it was so clear, we initially put the pSVV topic aside." (DiGA without pSVV_4)  "But if I look at the list of pSVV, then I wonder for 80% of the things, how am I supposed to prove that? And yes, how do I actually get that into studies?" (DiGA without pSVV_1) |
| Timing of the decision for endpoint varies | This theme emphasizes the centrality of proving medical benefits. The challenge of entering the DiGA market without them is significant. Guidelines prioritize clarity for evidence generation in medical benefits over pSVV. The focus on medical benefits stems from system acceptance and patient impact, highlighting their perceived higher quality. | "We have already provided digital care before there were DiGAs, and we have also gained experience with indications and patient groups suitable for DiGAs. So, the choice was based on experience and quickly made." (DiGA without pSVV_2)  "Well, those we have worked with a lot so far already had studies, and therefore, it was somehow always clear what we take. But I think these decisions, medical benefit versus positive structural and procedural improvement, are, I believe, made very early by most." (Consultant_6)  "We have already started thinking about clinical trials, at least this pilot study we are doing now, in broad strokes, in terms of the PICO schema, but the first line of code was still far from being written." (Health App_2)  "We decided very early on that we definitely want to improve the health status, improve the quality of life. So ideally, I make the decision before the development begins, as the entire development process needs to be built around this choice." (DiGA with pSVV_1)  "I think it varies a lot, but it is often quite late. So I can only say that from the perspective that we only support manufacturers because that is also one of our consulting areas, and they have problems or don't know much about it. But it can happen that manufacturers say, okay, we would like to submit the application now, but tell us again which questionnaire we should actually use or something like that. So it does happen very often." (Consultant_5) |
| BfArM has a strong influence on the selection of the endpoint(s) | This theme highlights selecting positive healthcare effects based on established measures. Manufacturers choose pVE guided by examples, and endpoints align with existing methods. pSVV complements when measuring instruments are available, simplifying the proof process. Integration of pSVV hinges on methodological availability, streamlining the assessment. | "But we will find it difficult to convince the authority that, if they have already well justified why they see it differently, it would be different. Unless we have different, so new information, but usually, one does not have that." (DiGA with pSVV_2)  "I would say that because the BfArM has the final decision-making authority on the question of what is being tested, I would already say that one is very much looking at what the BfArM says." (Regulatory Body_1)  "And then there are those who, so to speak, do it with the BfArM, as the BfArM actually wants it, in the consulting process beforehand and then almost set up the study with them. It is already the case that, of course, the BfArM says, well, you must know what you want to do and what you want to prove - in the end, what you prove must be plausible. How to prove that, the BfArM likes to talk about and says, but we need a different confidence interval, we need a different questionnaire, we need different endpoints, or we see, so to speak, what you have selected so far as methodologies, not suitable to prove the quality of life but rather patient sovereignty or something like that. So that means, in doubt, the categorization of what positive supply effect is shown changes." (Consultant_2)  "So there are very clear statements: 'Yes, this tool is suitable to prove X.' and later it says: 'No, but that is not suitable to prove that, then we need more...'" (Consultant_2)  "We were fortunate that we actually started with [Anonymized] when the DiGA guideline was already public. That means we knew what we were working towards, we knew in the annex which criteria, which requirements were coming our way." (DiGA without pSVV_1)  "So as a manufacturer, you would never do anything where the BfArM consultation said something else." (DiGA without pSVV_5)  "[The selection criteria are] essentially hard facts in the sense that it has already worked before, and there are validated measurement instruments, and then you take it with you. But if you would have to innovate the methods first and then also have the uncertainty that it could go wrong, the manufacturers are hesitant about that." (202306_Consultant_3) |
| Selection of endpoints based on established measuring instruments | This theme delves into the economic considerations in selecting positive healthcare effects. Study speed impacts planning, measuring instruments are chosen based on case requirements, study quality influences price negotiations, and seeking the "re-use" of pVE studies extends beyond the DiGA process. | "We started right away with pilot studies, more or less with small RCTs, and wanted to use the more or less highest standard for ourselves as a standard. We started with a relatively broad range of outcomes in various respects because, at the beginning, we knew nothing. The product is new, the setting was also new for us. As founders, we somehow never did an RCT before. So, this whole health services research is new, and therefore, we initially looked at what others had done." (DiGA without pSVV_1)  "There, you prefer to look for something that you can measure. Where there are validated questionnaires that the BfArM definitely accepts and already knows. And then you take the safe path with that." (Consultant_5)  "And then we checked, so we had the whole list, I think, six or seven, and then checked, what are the most important and probable, and then looked for measuring instruments for them. Based on the measuring instruments, we could determine the sample size needed, and based on the sample size, some were included or excluded." (DiGA with pSVV_1)  "I would say, if someone asked me from a Market Access perspective, study design, etc., what do you think? Should we include that? Of course, I would say, include it if it doesn't involve a lot of effort. But if it involves effort, I don't see the outcome that would be worth making an effort for it. Honestly, if we assume that we can achieve the medical benefit with the product, then it's secondary. Yes, but if it's not a big effort, I would say, include it. Yes, in the sense of the question. But it's said, I wouldn't base my strategy on it. I wouldn't say, do this, it's very, very important for us, and that's the most important thing." (DiGA with pSVV_2)  "So, there are ultimately a few questions, especially regarding health literacy; it is surveyed through questionnaires, patient safety as well, and patient sovereignty too. So, those are ultimately a few more questionnaires." (Health App_3) |
| Economic aspects of study conduction considered when choosing endpoint(s) | This theme underscores the significant influence of BfArM on selecting positive healthcare effects. Manufacturers closely adhere to BfArM advice, with DiGA guidelines serving as crucial guidance. BfArM's sway extends to influencing the choice of methodology in the selection process. | "And, ultimately, we paid attention to the implementation of the requirements of the BfArM— for example, their clear preference for certain aspects of documentation in the study protocol— as well as economic aspects, like the speed at which the study is conducted." (DiGA without pSVV_2)  "The study is set up in parallel, meaning the same positive effects are attested. One, of course, is the improved health condition through appropriate measures, such as outcome measures and interventions, quality of life, patient sovereignty, and health literacy. These are the four areas. The themes are health improvement and quality of life, both for DiGA and for the health insurance funds." (Health App_3)  "Do I want to have a medical benefit? Can I have a medical benefit at all? What does this potentially mean in terms of the scope of my study? Because I can also combine things there. I can conduct the study beforehand and then use it for medical evaluations for the medical product. For example, then I save myself double effort there." (Consultant_3) |
| **Dimension 3: Reasons for considering possibilities within new pathway** | | |
| New pathway used to generate additional endpoint(s) | This theme reveals the role of pSVV as an additional endpoint. It's relevant when combined with medical benefits, serving as a fallback option. Studied on the side for flexibility, its consideration is contingent on low effort, and it's non-confirmatory to manage costs effectively. | "So in reality, it is currently the case that it is somewhat always an additional care effect that one tries to address alongside the medical benefit. And also, to some extent, it might be a kind of emergency shelter if it doesn't fit with the medical benefits." (Consultant_4)  "Now this medical benefit is diluted by these things, which are all correct, important, and valuable, but which cannot be equated with medical benefits, and one does not want to equate them. There was already a great concern that this could be a softening of the gold standard, which one actually represents for the health insurance funds. So why we would say it is good, important, and right to consider this. However, fundamentally, there should always be evidence of a medical benefit. So the 'worst case' would be if this were considered independently. For example, if a DiGA could prove structural improvement, increased health literacy but might perform worse in terms of medical benefits, then one would say, yes, that is not sufficient. That cannot actually be. Therefore, one would say, a medical benefit is crucial, and structural and procedural improvement can additionally come into play. This can indeed be an enhancing criterion for the respective product. But it should not be considered independently of the hard fact of medical benefits, so to speak. But in the law, it is definitely equated." (Regulatory Body_2)  "I don't think the first focus was on the pSVV. I believe that, in fact, the medical aspect always took precedence. Yes, so the pSVV was, I assume, because it was predefined that there should be these two categories, people thought about it, but I believe the main focus was on the medical aspects." (DiGA with pSVV_2)  "But they didn't include it in the approval process because we wanted to keep the approval process streamlined, so we already somehow have only one primary endpoint that we test confirmatory. But in the protocol, in the entire study, we have also specified other additional analyses - secondary outcome points. And if they are effective, then we will submit a change request to the BfArM to include them retrospectively." (DiGA without pSVV_3)  "The BfArM is actually not interested in secondary outcomes." (DiGA without pSVV_1)  "[…] pSVV is included as a secondary endpoint in such a study. If possible, you confirm it, but you would always prefer to demonstrate medical benefits." (Consultant_3) |
| New pathway considered as a differentiation from the competition | This theme highlights pSVV's role in differentiation from competitors, serving as a distinctive criterion. Its significance is pronounced when distinguishing from the standard of care in the same indication, and the complexity of comparing DiGA underscores the importance of such differentiation. | "But in the case of [indication X], it could well be that with our DiGA, other DiGAs are also approved or provisionally approved. In such instances, I could envision that a pSVV might serve as a consideration for or against a DiGA." (DiGA without pSVV_4)  "I believe that it is important to have such effects to be able to justify a difference compared to others because the difference to other DiGAs ultimately gives a prescribing physician the opportunity to assess cost-effectiveness. So, there is a product that costs 200 euros and another one costs 210 euros. If they were exactly comparable, they would offer cost-effectiveness by saying you should choose the cheaper one. But if it has an additional feature and does something else, then, of course, you can deviate better, justify it better. In that sense, it has value." (Consultant_2) |
| Hope that considering new pathway can influence price positively | This theme illuminates the potential impact of pSVV on sales. Its importance lies in fostering acceptance among doctors and health insurance companies, enhancing visibility, and positively influencing patient perception, collectively contributing to its relevance for sales. | "So, one would say, medical benefit is the alpha and omega, and the structural and procedural improvement, that can additionally come into play. That can indeed be an enhancing criterion for the respective product." (Regulatory Body_2)  "Yes, I believe that in the last study we set up to be provisionally listed, we did it for reasons related to price negotiations that will eventually come up, so that you have another argument." (DiGA without pSVV_3)  "I believe that you negotiate it, so if you can demonstrate several medical or, sorry, if you can demonstrate several pVE, that can be quite useful in price negotiations. Although no one has shown yet that it goes higher with that. The arbitration board always comes to somewhat similar results. But that would be the wish for now." (DiGA without pSVV_2)  "So, they only have relevance if you have them as an add-on. Possibly for price negotiations." (Consultant_1)  "For the final price negotiations, it's different. The more evidence you have, the greater the likelihood of maintaining certain price points." (DiGA with pSVV_1)  "Possibly, pSVV can indeed be an enhancing criterion (during the price negotiation process) for the respective product." (Regulatory Body_2) |
| Consideration of new pathway can be relevant for sales | This theme explores the anticipation that pSVV holds relevance in pricing strategies. It is seen as a potential means to circumvent maximum price limits and considered as an additional factor in price negotiations, indicating the hope that pSVV can influence pricing outcomes. | "So, in our case, we actually go fully into a gap. And the patients simply wouldn't be treated otherwise. That means, the medical aspects are simply prominent, they stand out. But, as I said, in our case, the medical aspect is more in the foreground." (DiGA with pSVV_2)  "I believe that, fundamentally, it's something with which, even if it might not be firmly anchored in this DiGA structure, it is still a possibility that can be used to pull other players in the healthcare system more to one's side. I think that one can appeal very well to many doctors, perhaps also through one or another payer, and that it would be even better received if, let's say, the concept also thinks beyond that direction." (Health App_1)  “Distribution is also a crucial task we undertake when engaging with physicians, ensuring a clear delineation of what is effective beyond standard practices. Particularly with DiGA, the argument hinges on the fact that the pSVV dimension precisely provides the benefit." (DiGA without pSVV_3)  "But I do believe that it is better received by the patients. And of course, if a patient finds the app great and helpful, then the chance of them getting another prescription also increases. Accordingly, this might be an effect that should not be underestimated." (Consultant_5) |
| **Dimension 4: Hurdles for considering possibilities within new pathway** | | |
| Study design for new pathway difficult due to lack of measurement instruments (1/2) | This theme reveals the difficulty in designing studies for pSVV due to a lack of measurement instruments. The main hurdles include unclear measurement tools and methodology, a lack of standards for using real-world data, and challenges in operationalizing pSVV ideas, necessitating thorough discussions on methodological questions. | "I also believe, well, I think the fundamental flaw in this matter is that no one ever thought about how these things should be measured." (Regulatory Body_3)  "The counterpart in less established tools that are not exclusively validated for the disease might be different. And there's a bit of a difficulty in estimating that. What does that bring me? How can I deal with it?" (DiGA without pSVV_4)  "The methods are the problem, and the lack of hope, so to speak, that if you invest in it, it will work. We may focus more on what we know works." (DiGA without pSVV_2)  "And I wouldn't know how to measure that. Guidelines are guidelines, that means, it's not initially like a traffic regulation or something that is clearly defined, where you stop. Instead, it also leaves a room open and is more of a recommendation to the treating doctor. And how should I measure that through the DiGA? Especially because somehow I also have to capture a state at the start." (DiGA without pSVV_4)  "But when I look at them (the pSVV categories), I wonder with about 80% of them, how am I supposed to prove that? And yes, how do I actually incorporate that into studies? In the end, I have to put it into a study setting. Take the example of facilitating access to care. Where I think, yes, by offering the DiGA, I facilitate access to care. But that alone can't be a scientifically demonstrable construct or something." (DiGA without pSVV_1)  "Because, for one, this view is much more expressive, because, on the other hand, it is also undermined that the thing is actually being used. And that's a point. And therefore, I believe that there is currently a lack of a standard for these real-world data." (Consultant_3)  "I am convinced that there are simply too few established, validated measuring instruments for it, it's clear because it's completely new, there is nothing, and there is also nothing at the moment that is actually accepted in the market. And that's why it's super difficult to go there because, of course, if I say, yes, I'm improving the health condition with a 6-minute walk test, everyone says, yes, I know that, super. You have 30 meters more, perfect, full significance. If I now say, I have proven treatments according to the guideline. Everyone says, very nice. We have to do that too." (DiGA with pSVV_1)  "But I think this issue of measuring instruments, that there are too few validated measuring instruments, there is certainly a reason why it is also much more difficult to incorporate it as a secondary effect because for certain things, it is common with questionnaires, exactly, but not sufficient. And, a small shift, but for DiGA, for example, something in this direction will be needed because there is too little - medical benefits are simply - just reading through what needs to be done to validate these measuring instruments, you can already say, no manufacturer will do that." (Consultant_6) |
| Study design for pSVV difficult due to lack of measurement instruments (2/2) |  | "So my impression is actually, and that's why I'm a bit critical of the definition of the PSVV as it is now, that there was an attempt to arbitrarily throw in a few things that sound good and don't mean direct medical benefits, and there was also not much thought given to what extent this makes sense, to what extent we are only talking about a surrogate, and so on and so forth." (Regulatory Body_3)  "Yes, yes, so I also think that there was definitely too little real discussion around these method issues. This affects not only the methods for proof but also these arbitration models, for example, that I had mentioned. It's somehow like prices coming out of nowhere and it's basically being rolled, and it doesn't make much sense. That's why I'm there. So I also think there should be a larger expert round that really sits down with the fundamental questions there." (Regulatory Body_3)  "Yes, that's true. And I also believe that, as I said, if you go through the PSVV categories, then, I had already said that, then there are a few things where you say, yes, I can think of something right away, how I can operationalize that quite well. And you have a few things where you sit there and think, yes, that sounds really great, but you can maybe evaluate that from a Public Health perspective. Like facilitating access to care. No idea. And yes, so I think it's difficult. So I think it's good to somehow capture this benefit and process benefit." (Regulatory Body_3) |
| Lack of financial impact | This theme explores the "Lack of financial impact" associated with pSVV. It is unclear if pSVV influences doctors' decisions or price negotiations. The type of pVE detection is deemed irrelevant for patients, leading to the perception that pSVV entails effort without significant effects. Manufacturers face high uncertainty and costs, rendering pSVV financially unattractive. | "Purely from the procedural and financial perspective – if you were to ask our boss, who is responsible for finances – there is a clear answer: this is an absolute disaster." (DiGA with pSVV_1)  "Yes, so from the entire approval process, that is probably a relatively limited view. Financially, it's small. So, I currently don't see that it's worth much in the price negotiation to show that you have pSVV. I also see a big problem if a DiGA manufacturer really only comes into price negotiation with a pSVV because the acceptance for the DiGA and what the DiGA achieves will be even lower." (Consultant_5)  "I believe financially it is not the best case for manufacturers." (Consultant_2)  "But if it involves effort, I don't see the outcome that would be worth making an effort for it." (DiGA with pSVV_2)  "The methods are the problem, and the lack of hope, so to speak, that if you invest in it, it will work. We may focus more on what we know works." (DiGA without pSVV_2)  "Where it somewhat emerges is then again in the interpretation of the arbitration body, how do they take the pSVV into account? They also say in their arbitration decisions, yes, and furthermore, consider that there is the pSVV here in this case. But the actual trigger, so the arbitration model, there can be a lot of discussion about it, so it's more like rolling the dice, I would say. But the actual trigger that the arbitration body chooses is always the medical benefit. So they would never, or at least that's the experience, go the route of saying, yes, we are trying to base our price derivation essentially on the pSVV determined here." (Regulatory Body_3)  "It is indeed something fundamentally new, and I could imagine, as I said, you just mentioned it again, it is currently an absolute rarity for manufacturers to only focus on structural and procedural improvement. Probably, manufacturers had it in mind before that they need to conduct an RCT and focus on medical benefits, otherwise, their remuneration will be significantly lower because people will speak ill of it. So, this is already the anticipation that basically it is expected, otherwise, the economic result will probably be worse, although I must say, there is no evidence for that. So sure, one can take that standpoint, but the law does not allow for it, that with evidence, only a structural procedural improvement, the price would be severe or something like that. There is no basis for that in the case of Digas. In that regard, I see, by law, an equal footing." (Regulatory Body_2) |
| Method innovation not attractive for entre-preneurs | This theme highlights the reluctance of DiGA manufacturers towards method innovation. The perceived complexity of many steps, the increased expense associated with pSVV in study design, and the perceived high risk collectively diminish the attractiveness of adopting method innovations in this context. | "There are often no validated questionnaires or endpoints for this. The BfArM also accepts that it needs to be validated initially, but that is, of course, a process that becomes very difficult when I first have to validate the endpoint I want to achieve in the study." (Consultant_4)  "[The selection criteria are] essentially hard facts in the sense that it has already worked before, and there are validated measurement instruments, and then you take it with you. But if you would have to innovate the methods first and then also have the uncertainty that it could go wrong, the manufacturers are hesitant about that." (202306_Consultant_3)  "Oh, definitely not, because establishing a questionnaire, including validation and everything related, is not worth the effort." (DiGA with pSVV_2)  "But I believe this issue of measuring instruments, that there are too few validated measuring instruments, is certainly a reason why it is much more difficult to incorporate it as a secondary effect because, for certain things, it is common with questionnaires, yes, but not sufficient. To digress a bit, but for DiGAs, for example, something in this direction will be needed because there is too little - medically useful ones are simply - just reading through what needs to be done to validate these measuring instruments, one can already say that no manufacturer will do that." (Consultant_6)  "Yes, you need the validation of the measurement for that. You don't need control, but another measurement. For example, if you measure adherence through the app once and at the same time provide the questionnaire, then you have 2 statements because 2 measurement points for the same behavior. If they correspond or correlate, that is an indicator of the quality of the measuring instrument. [...] Or you do it simultaneously and even involve the perspective of others, for example, external evaluation by doctors or people from the patient's environment. The crucial thing is always that you have several measurements on the same topic, and if they correlate, then it is somehow documented that the basic behavior has actually occurred." (DiGA without pSVV_2)  "Yes, we also have such customers (who want to enter the market only with pSVV), but those are, I would say, the challenging projects. So, they then have an enormously hard time getting into the directory. Everything then becomes more difficult, designing the study, dealing with the BfArM, the discussions just become more difficult. Yes, but there are, of course, Use Cases for DiGA that purely focus on these pSVV. Yes. So, we don't see without reason hardly any or very few in the directory." (Consultant_5)  "If you had to essentially innovate methods and also deal with the uncertainty [...] in the worst-case scenario, you would end up with three studies. […] That alone costs you a million, just for these studies. No one is willing to foot that bill. And then, if it goes wrong..." (Consultant_3) |
| New pathway is currently not considered due to high uncertainty (1/2) | This theme exposes the current non-implementation of pSVV due to elevated uncertainty. Factors include evolving BfArM recommendations, the suggested but unassessed nature of the pSVV category, personal biases among BfArM employees, the need for clear communication to doctors and patients, and the absence of known price comparisons for pSVV. | "Thus, there is a discussion, and one could probably elaborate on it extensively whether a DiGA must have a medical benefit in the sense of the DiGA risk group or not. And I have the impression that the pendulum is increasingly swinging towards the need for one, with the reasoning being, well, it is a medical product, accordingly, it needs a Medical Purpose, it must also have a medical intended use, so it cannot be a medical product." (Health App_2)  "Yes, exactly, that was actually also in the conversation with the BfArM, our approach to say, yes, that is probably a reduction of, what is it called, the burden in everyday life for the patients and their relatives or something. And I am curious, yes, the BfArM said, yes, they could imagine, and then something surprising came, and I don't know, whether it will happen at the end of the day or not. The BfArM said, well, they could also imagine, because the app addresses the family as a whole system, that in the case, if a positive effect is seen in the child, which must definitely be seen, which is also correct because it is still about the child as a patient, that if one also sees a top, also with the parents and the option of the burden, that this can also be evaluated as part of the medical benefit." (Health App_2)  "ZL: Yes, exactly. As I said, we had actually already coordinated almost the entire study design with the BfArM. So, there were one or two rounds of discussions, also regarding which questionnaires, which is exactly the primary outcome or the secondary outcome. It was, I think, like with many others, quite a pain because we also received very difficult statements from the BfArM, where questionnaires were rejected that were accepted by other DiGA manufacturers. And these usual aches and pains. SD: And that is interesting. So, was it simply because somehow another case worker, or what do you think, how did it happen that it was accepted for others, and in your case, not? ZL: Yes, exactly, that is actually the only explanation we have." (Health App_1)  "With a break of 2 to 3 days, there was also a new question that I hadn't seen before. It is not announced that something like that suddenly happens. In this case, it did not lead to too much trouble, but it is sometimes challenging that the requirements change. The implications can be bigger or smaller. Yes, and thirdly, I believe the whole thing is crucial with which case worker you are dealing with. Regardless of the fact that in theory, everything should be universal and invariant, there are personal preferences and dislikes of the employees. And, as I have perceived it, there is also a lot of room for interpretation among the providers in the assessment of the same statements." (DiGA without pSVV_2)  "And I believe we are aware that it is also a learning system. And that the BfArM is maturing, so to speak. Yes, I believe the development is often like this: they discover an issue with a DiGA and then try to build a position on it, and if it happens a bit more often... that has been seen with some issues, then they eventually write it into the guideline to create transparency directly. I find that acceptable and also sensible. I think a big challenge is planning security or the lack of planning security in this regard. So, when we have coordinated all the questions, we answer them, and suddenly new ones come up, and we go another round..." (DiGA without pSVV_2) |
| New pathway is currently not considered due to high uncertainty (2/2) |  | "But, well, the question of what will be positively evaluated in a reimbursement negotiation. That is an indicator where one has to say it also has a slight push towards medical use and away from the pSVV. But I would say upfront, that is probably the question of what the BfArM is most likely to wave through, and since many manufacturers have gone into the area of medical use, one says, let's take what we know before venturing into new territories." (Regulatory Body_1)  "Well, that's just a very personal or where I think that could be the reason, the guideline was written not only by BfArM employees but also in collaboration with the BMG and consultations, I believe. Actually, you can see in the evaluation that the questions about the positive supply effect and the study design are actually evaluated by the established departments of the BfArM. So, for example, I don't know how the department of medicine or something, depending on the indication, you are assigned someone knowledgeable there who also does the consultations and who also has not only DiGA but usually drug studies on their desk. Yes, so that the peculiarities that may have been granted for digital applications are somewhat lost there at the BfArM, so to speak, in the corridors during the handover to these medical departments. So, that would be structurally the explanation for me. And I think that's what we notice. Not only with the study designs – we somehow also notice how certain parameters are measured. So, they have their drug goggles on first, and you have to convince them first that maybe it's a bit different here." (DiGA without pSVV_2)  "Well, I also think that there definitely was too little real discussion around these method issues. This concerns, on the one hand, the methods for proof, but on the other hand, of course, also these arbitration models, for example, that I mentioned." (Regulatory Body_3)  "Also, it is not quite so easy because they have very extensive expertise at the BfArM, but it is very drug-driven, so also geared towards drug studies. And you often hear, so in drug studies it is like this and that, but we are now in the DiGA area, so it is different. The problem, however, is that in people's minds, these evidence criteria and the criteria from drug studies are very ingrained, and it is difficult to soften them." (DiGA with pSVV_1)  "There is a lot of need for explanation, actually, for the different ones. Both with the patients and with the doctors. What is it anyway? Why is it good? What does it bring me? With the doctors, I have to respond to that. That is an additional task for me. I make sure that I report to the patients more often. It takes away patients from me because they don't report anymore. It interferes with my therapy. Things like that have an extreme impact. So, we have to invest an incredible amount of time in marketing, communication, education, acceptance." (DiGA with pSVV_1)  "So, and since the risk for the primary endpoint is so high that no one who can show anything medically will show pSVV, it is actually a dead end." (DiGA without pSVV_5)  “There is a significant need for explanation, actually, among the different stakeholders. Both with patients and with doctors. What is it exactly? Why is it good? What is in it for me?” (DiGA with pSVV_1)  "No, with this construct of positive structural and procedural improvement, patient-relevant structural and procedural improvement - not that they (BfArM) fundamentally reject it, but we experience that they, how should I say, are not so sure about it. So, they struggle with it." (Consultant_5) |
| Consultants advise against new pathway | This theme reveals a trend where consultants discourage pSVV. The preference leans towards mN due to familiarity. Permanent acceptance of pSVV is hindered by the requirement for concurrent medical benefits, aligning with BfArM's insistence and the prevailing system understanding centered around medical benefits. | "Well, we unfortunately have to advise to somehow rely on the medical benefit. That is also a bit your issue because the BfArM seems to have some problems with it. No, with this construct of positive structural and procedural improvement, patient-relevant structural and procedural improvement - not that they fundamentally reject it, but we experience that they, how should I say, are not so sure about it. So, they struggle with it." (Consultant_5)  "We even supervised an indication where the BfArM says, there is only the medical benefit. So, we cannot decide on any pSVV, that is impossible." (Consultant_4)  "And there were similar statements from others as well. So, there were many statements that were extremely negative towards the symptoms, structural, and procedural improvement. So, we would say, if you believe you can demonstrate medical benefits, that is probably the better path towards price negotiations because it's something the system understands and can perhaps evaluate better." (Consultant_6) |
| **Dimension 5: Targeted innovative technology important for the healthcare system** | | |
| Innovative technology focuses on additional effects for the system (1/2) | This theme highlights how pSVV centers on providing additional benefits for the system. It aims to address the strain on individuals within the system, enhance care processes, collect supplementary information for practitioners, and extend patient support beyond health improvement, addressing a perceived gap in available therapies. | "The idea of pSVV, I don't know if it really comes into play as such. Because with it, you can somehow address low-risk, low-threshold offerings, softer criteria. I actually think the idea is really good, especially when it comes to patient autonomy, quality of life, and processes that get lost in this mass of information." (DiGA without pSVV_4)  "But if we now say, we would compare ourselves to a classical intervention [...] then the pSVV [...] plays an even greater role. Because if I say, I have something here now, and it has a positive effect and is more accessible than therapy, that is already a value where I say, that makes the difference." (DiGA with pSVV_2)  "Because it is also relatively obvious that digital applications often have a rather process-changing character. And that it is not always about directly influencing physiological processes or something like that, and then primarily achieving patient benefit through an improvement in medical health, but rather because somehow the care processes, which may not work perfectly at some points and could be improved, are tried to be improved." (Regulatory Body_3)  "Well, as a health economist, I would fully agree that somehow a reduction in travel times or that it is fundamentally a relief for patients and relatives is a benefit." (Regulatory Body_3)  "The patient feels subjectively more capable of making health decisions. Probably has an influence in general. It is also interesting for the payer because such patients usually perform better with interventions since they understand it more and feel they are more in control." (Consultant_1)  "I believe that you, of course, have the chance to address other care effects that may not be addressable with corresponding technologies." (Consultant_4)  "All providers actually signal to us that adherence is the big problem." (DiGA without pSVV_2)  "If my system, however, does not allow improving the health status, then I at least have the opportunity to help the patient in other ways." (Consultant_4)  "When this concept of benefit was further developed, maybe also with the idea in mind that DIGAs should essentially help the patients, strengthen their role, also in the doctor-patient relationship, that they are better informed about their illness, know what's going on, and can actively contribute to treatment outcomes, more actively than perhaps uninformed patients, so that, through DIGAs, there is essentially a form of knowledge transfer." (Regulatory Body_2)  "And I believe that a fundamental understanding also needs to be created that this is also a care effect that is meaningful for patients and improves care. And not just a secondary endpoint in the studies and also a secondary care effect, but it is just as much a primary effect as perhaps the quality of life is." (Consultant_4) |
| Innovative technology focuses on additional effects for the system (2/2) |  | "It also makes sense from the overall healthcare system perspective. Because then we actually have what we always say so nicely in some political discussions as efficiency improvement, and we actually have it. Yes. But that is a completely different example than, for example, taking the point of adherence." (Health App_2)  "And then also towards the sales team, that is also an important task we have when talking to doctors, that we can clearly name what works and is outside the norm. And especially with DiGAs, the argument is that through the pSVV dimension, you have the benefit." (DiGA without pSVV_3)  "And I think, when this concept of benefit was further developed, maybe also with the idea in mind that DIGAs should essentially help the patients, strengthen their role, also in the doctor-patient relationship, that they are better informed about their illness, know what's going on, and can actively contribute to treatment outcomes, more actively than perhaps uninformed patients, so that, through DIGAs, there is essentially a form of knowledge transfer." (Regulatory Body_2)  "And about that, I think, it has played a bit of a role, this patient-relevant structural, procedural improvement. These are things like patient sovereignty, health literacy, better access to the system, which one probably also had in mind. And with DIGAs, many data are ultimately collected, which, when it all works together with the ePA, can also be taken across sector boundaries, so that the healthcare providers in the hospital are even better informed about the information, data that I may have collected and generated with the DiGA." (Regulatory Body_2)  "Well, because I believe that the healthcare system often does not suffer from the fact that there is no way to be treated, but that people are overwhelmed with the way it works, how the individual services are coordinated, how one understands, how it is told to you in healthcare, how one classifies it, how one implements it. And at such points, there is a lot of better care inside." (Consultant_2) |
| Innovative technology relevant for patients | This theme underscores the relevance of pSVV for patients. It is designed to showcase the empowerment of the patient's role, and its impact extends to reaching patients in their everyday lives, emphasizing the significance of pSVV in patient-centric contexts. | "And when he (the patient) stops with it (using the DiGA), so when he is no longer digitally supported, it is also quite interesting and very clearly demonstrable that it immediately, so it deteriorates again, and he will have more decompensations again." (DiGA with pSVV_1)  "Well, I think they wanted to expand it because it is also relatively obvious that digital applications often have a rather process-changing character. And that it is not always about directly influencing physiological processes or something like that, and then primarily achieving patient benefit through an improvement in medical health, but rather because somehow the care processes, which may not work perfectly at some points and could be improved, are tried to be improved." (Regulatory Body_3)  "Well, it does play a role, it is not insignificant, because the waiting times are already very long. And until you find a proper doctor or psychotherapist who suits you, too. And this problem can be avoided, and I already find that is a great value of DiGAs. Not just the medical aspect." (DiGA with pSVV_2)  "Exactly, that's also a point, people always like to cling to these medical benefits, where I say, if I now increase adherence and health literacy in a patient, and as a result, for example, the blood pressure also plays out better because he takes his pills, I see that as a form of medical benefit, at least supportive." (Consultant_3)  "You just say hey, the patient feels subjectively more capable of making health decisions. Probably has an influence in general - it is also totally interesting from the payer's perspective - because individuals with higher health literacy typically respond more effectively to interventions, as they have a better understanding and feel a greater sense of control." (Consultant_1)  "So what I believe, what I see as a really big chance from these patient-relevant structures, procedural improvements. And I think that is, I believe, underestimated too, I just measure the patients' everyday life with it. I don't necessarily always get that with these medical benefits. Because when patients are at the doctor's, it's always a situational recording, and either the patients are nervous and therefore worse at the doctor's, or they are positively excited, and then they are better, so they are better or worse than at home. But 80 or 90 percent of the time, they are alone at home, and I can't assess and evaluate that. And with this, I can already assess and demonstrate it and see the real reality, how it really is." (DiGA with pSVV_1) |
| Special benefits of Digital Health Applications are lost in the classic benefit assessment | This theme highlights the loss of DiGA's unique advantages in the conventional benefit assessment. Technologies challenging to assess via mN find expression in pSVV, illustrating DiGA's distinct benefits compared to drugs and revealing positive effects across the entire system. | "So, I believe we would very vehemently state that DiGA can have medical benefits, but they are not solely limited to medical utility. But honestly, they also have capabilities that are listed in the DiGA guideline we wrote, such as adherence, relief of treatment processes, and these are points where they can contribute to health applications. And these are points that get lost in traditional benefit assessments." (Regulatory Body_1)  "One might also need to consider this concept of benefit, which has always been a big point of discussion before. It was said here that, yes, you cannot capture the benefit of a digital application only through medical benefits, but other things must also be taken into account." (Regulatory Body_3)  "Actually, all providers signal to us that adherence is the major problem." (DiGA without pSVV_2)  "I believe that you naturally have the opportunity to address other therapeutic effects that may not be addressable with corresponding technologies. [...] I certainly have the chance to make a significant contribution to improving care, which I wouldn't have if I only address quality of life and health status." (Consultant_4)  "pSVV is crucial for the system, but the evidence must focus on it. Thus, the usability of the product must be ensured and tailored to that. The evidence must reflect the particular added value of these applications." (DiGA without pSVV_5)   "But if we now say we would compare ourselves to a traditional intervention [...] then the pSVV [...] plays an even greater role. Because when I say, I have something here with a positive effect, and it is more accessible than the therapy, that is already a value where I say, it makes a difference." (DiGA with pSVV_2)  "And especially with DiGAs, the argument is that through the pSVV dimension, you have precisely the benefit." (DiGA without pSVV_3)  "Yes. But it also makes sense, not just for services, but it also makes sense from the overall health system perspective. Because then we actually have what we always talk about so nicely in some political discussions as efficiency improvement, and we actually have it." (Health App_2) |
| Innovative Technology politically wanted and inter-disciplina-rily developed | This theme reveals that pSVV is politically desired and collaboratively developed. Rooted in HTA dimensions with a patient focus, the concept expanded through collaborative efforts, influenced by international examples like France's extended utility concept, and emerged from a broader working group perspective. | "We had many stakeholders involved there. On the one hand, we had colleagues from self-administration, but we also had the manufacturers, combining forces [...] in understanding potential, addressing challenges, and bridging the gap between traditional assessment methods and innovative logics.” (Regulatory Body_1)  "What are actually the positive structural and procedural improvements? They are somewhat derived from other HTA dimensions that are floating around. So, they didn't come out of nowhere." (Regulatory Body_1)  "This (pSVV) can also be a valorizing criterion for the respective product." (Regulatory Body_2)  "Well, I think that was intended to be expanded because it is also quite obvious that digital applications often have a character of process change. And it's not always about directly influencing physiological processes or something like that and then primarily benefiting the patient by improving the medical health condition, but rather, because one somehow tries to improve the care processes that may not work perfectly at some points." (Regulatory Body_3) |

Table 3 - Selected codes generated from interviews using grounded theory

## Functional-Structural Analysis of pSVV as a Systemic Innovation from Concepts generated in Qualitative Analysis

| **Structural Elements** | **Actors** | **Institutions** | **Infrastructure** | **Interactions** | **Functions Evaluation** | **Description of problems** | **Reasons why the specific function is absent ('blocking mechanism')** | **Systemic problem** |
| --- | --- | --- | --- | --- | --- | --- | --- | --- |
| **Functional elements** |  |  |  |  | **Scale: 0=absent, 1=very weak, 2=weak, 3=moderate, 4=strong, 5=very strong** |  |  |  |
| **F1 - Entrepreneurial activities** |  |  |  |  | 1 |  |  |  |
| - Are there enough entrepreneurs? - What is the quality of entrepreneurship? - What types of businesses are involved? - What are the products? - To what extent do entrepreneurs experiment? - What variety of technological options are available? - Are any entrepreneurs leaving the system? - Are there new entrepreneurs? | Manufacturers: - Medical benefits also in focus independent of DiGA - “Re-use” of the pVE study is also sought outside of the DIGA process - pVE and DiGA process (and therefore pSVV) new for manufacturers who have been on the market for a long time - pVE is chosen before the product is developed - Decision pVE often only takes place shortly before the application to the BfArM | Established practices:  - Medical benefits also in focus independent of DiGA |  |  |  | There are no manufacturers currently active in the market whose product focuses specifically on one of the pSVV categories. | Actors have been in the market longer than pSVV exist and therefore have focused their product on medical benefit and/or Actors try to combine efforts towards evidence generation with other ways of market access that do not know the pSVV concept (e.g. "Selektivverträge") | Actors’ presence problem - No companies with focus on pSVV |
| **F2 - Knowledge Development** |  |  |  |  | 3 |  |  |  |
| - What is the knowledge base in terms of quality and quantity? - Is the knowledge basic or applied? - Are there many projects, research, patents and articles? - Is there a leading international position, trigger programs, many cited patents? - Which actors are particularly active? - Who finances the knowledge development? - Does the technology receive attention in national research and technology programs? - Are there enough knowledge users? |  | Instructions: - DiGA guidelines clear for medical benefits - Clarity of evidence greater for mN than for pSVV - DiGA guidelines provide important guidance for choice of pVE | Standards:  - Endpoints are chosen according to existing methods - Proof of pSVV is not complex if questionnaire is available |  |  | The current knowledge about pSVV is limited as there is no example already in the market to compare to. Standards are missing especially regarding accepted methods to proof pSVV -> Infrastructure problem | No products in the market to learn from; Suitability of available methods for pSVV products limited | Infrastructure presence problem - Knowledge about concept of pSVV limited |
|  |  | Instructions:  - pSVV good ideas but difficult to operationalize- Definition and proof of pSVV not thought through | Knowledge:  - The entire system only understands medical benefitsStandards: - Lack of methods is the biggest hurdle for proof of pSVV- There is no standard for using real world data |  |  | While there was a DiGA guideline published to further specify the DVG and the respective DiGA Ordinance, these Instructions are not deemed sufficient as they are more specific for medical benefits and hard to operationalize for pSVV -> Institutions problem | DiGA Guideline is more specific on medical benefit than on pSVV. Exemplary topics for pSVV are defined but no examples for methodology | Institution intensity problem - Standards missing in DiGA Guideline |
| **F3 - Knowledge Dissemination** |  |  |  |  | 1 |  |  |  |
| - Are there strong partnerships? - Between whom? - Is the knowledge development demand-driven? - Is there space for knowledge dissemination? - Is there strong competition? - Does the knowledge correspond with the needs of the innovation system? - Have any licenses been issued? |  |  |  | Network level:  - pVE are chosen based on the example of other manufacturers  'Individual level:  - Manufacturers do not deviate from recommendations from BfArM advice - BfArM has a strong influence on the choice of methodology |  | Interactions with BfArM as advisor within the process are strong and partially contradicting use of pSVV due to stronger experience with medical benefit -> Actors/Interaction problem  Knowledge dissemination was mentioned in terms of looking to other manufacturers for guidance and examples. As very few have used pSVV so far, the Knowledge Dissemination in this area is very weak. Other Actors are not active -> Actors/Interaction problem | Weak knowledge dissemination via multiplicators due to powerful focus on medical benefit in combination with low interaction between manufacturers due to lack of experience | Interactions intensity problem - Strong interactions towards medical benefit |
|  | BfArM: - BfArM comes from a pharmaceutical mindset  Other stakeholders: - It is unclear whether pSVV is relevant for decision-making by doctors - Type of pVE detection is irrelevant for patients - There is a great need for pSVV to explain this to doctors and patients |  |  | Network level: - Discussion on the pSVV method question required |  | The BfArM in its role as advisors for manufacturers wanting to go through the admission process also has no experience with pSVV but rather a strong focus on medical benefits coming from a pharmaceutical background.  Other stakeholders, specifically practitioners supposed to prescribe the product as well as users of the product do not know pSVV yet and do not express demand specifically. | The supervising governmental institution, in its role as advisor for manufacturers in the admission process, has no experience with pSVV but rather a strong focus on medical benefits coming from a pharmaceutical background. Prescribing practitioners as well as users of the products have limited experience with pSVV focusing products and do not specifically demand them. | Actors’ capability problem - Knowledge multiplicators as well as other stakeholders with limited experience with the pSVV concept |
| **F4 - Guidance of the search** |  |  |  |  | 2 |  |  |  |
| - Is there a clearly articulated and shared goal for thesystem? - Is it generic or specific? - Is it supported by specific programs, policies, who arethe system’s frontrunners? - Is the objective inducing government activities? - What are the technological expectations (negative/positive)? - Does the articulated vision fit in the existinglegislation? |  | Established practices:  - Difficult to enter the market for DiGA without medical benefits- pSVV rather as an addition to mN if the measuring instrument happens to be available | Knowledge:  - pSVV only if methodology is available |  |  | Goal of introducing products that apply to pSVV categories is articulated in DiGA guidelines but there are points that contradict it:- Established practices during acceptance process are not in line with introduced pSVV policy -> Institutions problem | Established practices during admission process not in line with DiGA Guideline, BfArM insists on medical benefits | Institution intensity problem - Established practices hamper the establishment of pSVV |
|  |  | Established practices: - Subsequent changes by the BfArM (recommendations and Guideline) - pSVV category suggested but still med. Benefits assessed - pSVV currently not accepted permanently without med. Benefits  - BfArM insists on medical benefits - BfArM employees have personal likes and dislikes | Standards: - Birth defects of pSVV are unclear measurement tools and methodology - No comparison prices for pSVV are known |  |  | There are standards and knowledge missing in the policies regarding pSVV -> Infrastructure problem | Missing standards for methods to measure pSVV lead to limited adoption. Existing methods to measure therapeutic effects are rarely suitable for pSVV and missing positive price examples limit support of manufacturers | Infrastructure presence problem - Knowledge about concept of pSVV limited |
| **F5 - Market formation** |  |  |  |  | 1 |  |  |  |
| - What does the market look like? - What is its size (niche/developed)? - Who are the users (current and potential)? - Who takes the lead (public/private parties)? - Are there institutional incentives/barriers to market formation? - Must a new market be created or an existing one be opened up? |  |  |  | Consultants/Companies: - Push towards mN – you take what you know |  | Manufacturers who use pSVV do not participate to market formation of products that address pSVV categories but rather use pSVV as an addition to proving medical benefit as a fallback or for flexibility, hence a market for products focusing on products addressing pSVV categories has not been formed yet -> Actors problem | No product in the market with specific focus on pSVV; pSVV rather used in combination with medical benefit | Actors’ presence problem - No companies with focus on pSVV |
|  | Manufacturers:  - pSVV only relevant in combination with medical benefits- pSVV as a fallback if medical benefits do not work- pSVV is also collected for flexibility | Established practices:- pSVV not confirmatory in order to keep costs low (Secondary outcomes are not the focus of the BfArM) |  |  |  | Barriers induced by interactions with consultants that push towards using medical benefit slow down market formation -> Interactions problem | Strong interactions with consultants that push towards medical benefit | Interactions intensity problem - Strong interactions towards medical benefit |
| **F6 - Resources mobilization** |  |  |  |  | 1 |  |  |  |
| - Are there sufficient financial resources for system development? - Do they correspond with the system’s needs? - What are they mainly used for (research/application/ pilot projects etc.)? - Is there sufficient risk capital? - Is there adequate public funding? - Can companies easily access the resources? | Companies:  - Speed of study implementation relevant for planning | Established practices: - pSVV is only considered if there is little effort - Selection of measuring instruments depends on the number of cases required - Quality of the study is assessed as relevant to price negotiations |  |  |  | The inclusion of pSVV in the list of possible positive healthcare effects (DVG -> DiGA Guide) made financial resources in terms of access to statutory reimbursement available for products focusing on pSVV categories. Though, high cost for development of innovative methods to measure pSVV effects contradict this possibility. The manufacturers (all SMB) are cost conscious in their choice of study design, pSVV is only considered if there is limited effort to be expected. In combination with several hurdles to be expected in terms of high cost for innovative method design and limited financial gains that have been proven so far, the use of pSVV is deemed to high risk -> Institutions problem | DiGA Guideline not specific enough on pSVV - neither examples nor exemplary endpoints - to avoid costly method innovation for manufacturers | Institution intensity problem - Standards missing in DiGA Guideline |
|  |  | Expectations:  - It is unclear whether pSVV is relevant for price negotiations- High uncertainty for manufacturers combined with high costs- pSVV is not financially attractive for manufacturers- Method innovation requires many steps- pSVV makes study design more expensive- Method innovation too much risk- pSVV is effort without effect |  |  |  |  |  |  |
| **F7 - Creation of legitimacy** |  |  |  |  | 2 |  |  |  |
| - Is investment in the technology seen as a legitimatedecision?¢ Is there much resistance to change? - Where is resistance coming from? - How does this resistance manifest itself? - What is the lobbying power of the actors in thesystem? - Is coalition forming occurring? |  | Established practices: - Medical benefits are perceived as being of higher quality- The focus is on medical benefits because it makes the most sense for patients |  |  |  | Currently the customs during the process of choosing the positive healthcare effect are leaning towards medical benefit, indicating that using pSVV is not perceived as a legitimate decision.  Positive expectations are related to improvement of care in the system as well as positive effects on distribution. Though economically relevant claims for manufacturers are currently hypothetical.  The legitimacy for these expectations party derive from the knowledge around how the concept of pSVV have been developed and interactions on an international level.  It is to say that the current customs are stronger than the positive expectations that would create legitimacy for use of PSVV -> Institutions problem | Positive expectations are related to improvement of care in the system as well as positive effects on distribution. Though economically relevant claims for manufacturers are currently hypothetical. Established practices are stronger than positive economic expectations and weaken the creation of legitimacy | Institution intensity problem - Established practices hamper the establishment of pSVV |
|  |  | Expectations: - pSVV possibly relevant for circumventing maximum price limits - pSVV may be relevant for price negotiation as an add on - DiGA are difficult to compare with each other - pSVV can be important for acceptance among doctors - pSVV may be relevant for acceptance by doctors and health insurance companies - pSVV can be good for visibility - pSVV relevant for patient perception - pSVV as a criterion for differentiation - pSVV is particularly important for differentiating from standard of care for the same indication |  |  |  |  |  |  |
|  |  | Expectations:-  (pSVV can help) addressing excessive demands on people in the system- Improve care processes- Collection of additional information for practitioners- Patients help beyond improving their health- Enough therapies but no support- pSVV reaches patients in everyday life- pSVV show the special benefits of DiGA- pSVV is an attempt to show the different benefits of DIGA and drugs- pSVV makes positive effects for the entire system visible- Technologies that are difficult to address via mN | Knowledge:  - Expansion of the concept of benefit emerged from an overarching working group- pSVV is intended to demonstrate strengthening of the patient's role- pSVV derived from HTA dimensions with patient focus | Network level:  - France has also expanded the concept of utility |  |  |  |  |
|  |  |  |  |  |  |  |  |  |

Table 4 - Functional-structural analysis of pSVV as a systemic innovation from concepts generated from theme (2)-(5) in the qualitative analysis

## Derivation of Strategic Factors from Systemic Problems Using the Functional-Structural Framework of Wieczorek & Hekkert^2^

|  | **Systemic Problem** | **Reasons why the specific function is absent ('blocking mechanism')** | **Instrument goal - Solution*** | **Example Instruments*** | **Strategic factor** |
| --- | --- | --- | --- | --- | --- |
| **F1 - Entrepreneurial activities** | Actors’ presence problem - No companies with focus on pSVV | Actors have been in the market longer than pSVV exist and therefore have focused their product on medical benefit and/or Actors try to combine efforts towards evidence generation with other ways of market access that do not know the pSVV concept (e.g. "Selektivverträge") | Stimulation of participation of manufacturers that build products with primary focus on pSVV | Public debates; Clusters; Venture Capital | Further stimulation of market formation |
| **F2 - Knowledge Development** | Infrastructure presence problem - Knowledge about concept of pSVV limited | No products in the market to learn from; Suitability of available methods for pSVV products limited | Stimulation of a knowledge infrastructure around pSVV | Classical R&D grants; taxes; loans; public research labs | Specification of goals and standards |
| **F2 - Knowledge Development** | Institution intensity problem - Standards missing in DiGA Guideline | DiGA Guideline is more specific on medical benefit than on pSVV. Exemplary topics for pSVV are defined but no examples for methodology | Stimulation of development/strengthening of instructions | Regulations; Norms; limits; obligations | Specification of goals and standards |
| **F3 - Knowledge Dissemination** | Interactions intensity problem - Strong interactions towards medical benefit | Weak knowledge dissemination via multiplicators due to powerful focus on medical benefit in combination with low interaction between manufacturers due to lack of experience | Stimulation of constructive exchange about pSVV | Political tools (Awards and honors for innovation development); demonstration centers; Constructive technology assessment; technology promotion programs; debates, discourses, venture capital | Further stimulation of market formation |
| **F3 - Knowledge Dissemination** | Actors’ capability problem - Knowledge multiplicators as well as other stakeholders with limited experience with the pSVV concept | The supervising governmental institution, in its role as advisor for manufacturers in the admission process, has no experience with pSVV but rather a strong focus on medical benefits coming from a pharmaceutical background. Prescribing practitioners as well as users of the products have limited experience with pSVV focusing products and do not specifically demand them. | Creation of space for capability development of stakeholders in the system | road-mapping; brainstorming; education and training programs; scenario development workshops; policy labs; pilot projects | Active management of innovation process |
| **F4 - Guidance of the search** | Institution intensity problem - Established practices hamper the establishment of pSVV | Established practices during admission process not in line with DiGA Guideline, BfArM insists on medical benefits | Avoiding too much focus on established practice (med. benefit) | Regulations; Norms; limits; obligations | Active management of innovation process |
| **F4 - Guidance of the search** | Infrastructure presence problem - Knowledge about concept of pSVV limited | Missing standards for methods to measure pSVV lead to limited adoption. Existing methods to measure therapeutic effects are rarely suitable for pSVV and missing positive price examples limit support of manufacturers | Stimulation of a knowledge infrastructure around pSVV | Classical R&D grants; taxes; loans; public research labs | Specification of goals and standards |
| **F5 - Market formation** | Actors’ presence problem - No companies with focus on pSVV | No product in the market with specific focus on pSVV; pSVV rather used in combination with medical benefit | Stimulation of participation of manufacturers that build products with primary focus on pSVV | Public debates; Clusters; Venture Capital | Further stimulation of market formation |
| **F5 - Market formation** | Interactions intensity problem - Strong interactions towards medical benefit | Strong interactions with consultants that push towards medical benefit | Stimulation of constructive exchange about pSVV | Political tools (Awards and honors for innovation development); demonstration centers; Constructive technology assessment; technology promotion programs; debates, discourses, venture capital | Further stimulation of market formation |
| **F6 - Resources mobilisation** | Institution intensity problem - Standards missing in DiGA Guideline | DiGA Guideline not specific enough on pSVV - neither examples nor methods - to avoid costly method innovation for manufacturers | Stimulation of development/strengthening of instructions | Regulations; Norms; limits; obligations | Specification of goals and standards |
| **F7 - Creation of legitimacy** | Institution intensity problem - Established practices hamper the establishment of pSVV | Positive expectations are related to improvement of care in the system as well as positive effects on distribution. Though economically relevant claims for manufacturers are currently hypothetical. Established practices are stronger than positive economic expectations and weaken the creation of legitimacy | Avoiding too much focus on established practice (med. benefit) | Regulations; Norms; limits; obligations | Active management of innovation process |
| *** Directly aligned with the proposed goals and instruments for specific systemic problems from Wieczorek, Anna J., and Marko P. Hekkert. 2012. ‘Systemic Instruments for Systemic Innovation Problems: A Framework for Policy Makers and Innovation Scholars’. Science and Public Policy 39(1):74–87. doi: 10.1093/scipol/scr008** | | | | | |

Table 5 – Derivation of strategic instruments to address identified systemic problems in alignment with functional-structural analysis framework^2^, clustered into actionable strategic factors

# Glossary

BfArM = Bundesinstitut für Arzneimittel und Medizinprodukte = Federal Institute for Drugs and Medical Devices

DiGA Directory = Directory of reimbursable digital health applications

DiGA Guide = DiGA Leitfaden

DVG = Digital Healthcare Act

mN = Medizinischer Nutzen = Medical Benefit

pSVV = Patientenrelevante Struktur- und Verfahrensverbesserung = Patient-relevant improvement of structure and processes

pVE = Positiver Versorgungseffekt = Positive Healthcare Effect

TIS = Technology Innovation System

# Additional remarks

The full COREQ Checklist for reporting qualitative studies is available upon on reasonable request.

# Bibliography

1. Federal Institute for Drugs and Medical Devices (BfArM). DiGA Directory of the BfArM. https://diga.bfarm.de/de/verzeichnis (2024).

2. Wieczorek, A. J. & Hekkert, M. P. Systemic instruments for systemic innovation problems: A framework for policy makers and innovation scholars. *Sci Public Policy* **39**, 74–87 (2012).
